# China’s growing influence in the global carrageenan industry and implications for Indonesia

**DOI:** 10.1007/s10811-023-03004-0

**Published:** 2023-06-08

**Authors:** Jing Zhang, Scott Waldron, Zannie Langford, Boedi Julianto, Adam Martin Komarek

**Affiliations:** 1 School of Agriculture and Food Sciences, University of Queensland, Brisbane, Australia; 2 The Australia-Indonesia Centre, Universitas Hasanuddin, Makassar, Indonesia; 3 PT Jaringan Sumber Daya, Makassar, Indonesia

**Keywords:** China, Indonesia, Carrageenans, Seaweeds

## Abstract

China has reconfigured the global value chains of a wide range of commodities. This includes carrageenan, a polysaccharide extracted from specific types of red seaweeds used as a gelling and thickening agent in a wide range of applications. In the past 20 years, China has moved to centre stage in the global carrageenan processing sector, with wide-ranging implications for seaweed producing nations and farmers. This is especially the case for Indonesia, a pivotal carrageenan seaweeds producer that exports almost all seaweed to China, cemented by large Chinese investments in processing in Indonesia. Despite the importance, there is a dearth of studies on the Chinese domestic industry and associated trade and investment flows. This study fills the gap by triangulating a range of detailed industry, statistical and interview data, in multiple language sources. It finds that Chinese trade and investment linkages is of net benefit to Indonesia but that Indonesian government agencies at both central and local levels can begin to introduce terms in their favour.

## Introduction

Carrageenan is a linear sulphated polysaccharide family extracted mainly from members of *Kappaphycus* and *Eucheuma*, two related genera of red seaweeds known collectively as carrageenan seaweeds (Valderrama et al. 2013), carrageenan-bearing seaweeds (Bixler and Porse 2011; Hurtado et al. 2017; Porse and Rudolph 2017) or eucheumatoids (Ali et al. 2017; Loureiro et al. 2017; Neish et al. 2017). Carrageenan is utilized extensively as thickener, stabilizer or gelling agent in a vast array of processed foods, cosmetics, pharmaceuticals and biotechnological applications (Necas and Bartosikova 2013; Bui et al. 2019). The industry had a turnover of US$871.7 million in 2022 which is expected to reach US$1.3 billion by 2030 with burgeoning demand for processed, convenience or organic food from consumers worldwide (Grand View Research 2023). The carrageenan processing sector was traditionally dominated by multinational corporations from “Western” countries (the US and in Europe) including through direct foreign investments in the Philippines (Richards-Rajadurai 1990; Blanchetti-Revelli 1997; Neish et al. 2017; Palanca-Tan 2018; BFAR 2022). Driven by the technological advancement, companies from China have risen from a low base to play a growing and perhaps dominant role in the sector as a processor, trader, market and source of outward investment (Bixler and Porse 2011; Campbell and Hotchkiss 2017; Hurtado et al. 2019).

The rising prominence of Chinese companies in the downstream sectors of the global carrageenan industry could have far-reaching implications. Firstly, China's ascent as the world's largest processor of carrageenan-bearing seaweeds, and the associated structural industry changes and outward investment patterns has the potential to significantly influence the derived demand for raw seaweed and the prices that small coastal seaweed farmers receive for their product (Blanchetti-Revelli 1997; Porse and Rudolph 2017). Secondly, Chinese outward investment provides opportunities and threats for policymakers in destination countries interested in industry development and generating employment and tax revenues, but who are also subject to pressure from domestic industry interests. Third, large corporate interests in traditional western countries are very interested in the rise of Chinese carrageenan processors – either as competitors or as partners. Moreover, China's growing influence in the global governance of trade, food safety and environmental protection may have significant and wide-ranging implications for the entire carrageenan industry (Chan et al. 2011; Liu et al. 2019; Coenen et al. 2021; Dong and Li 2021).

Nowhere are these implications more direct than for Indonesia. Indonesia is the world’s largest producer of carrageenan seaweeds (Hatch Seaweed Insights 2023a). Trade statistics report that the vast majority of carrageenan seaweed exports from Indonesia was shipped to China (UN Comtrade 2022). In addition, the three largest hydrocolloids processors in China – and a range of other processors – have invested heavily in the Indonesian processing sector, to now account for nearly half of all domestic processing throughput (Komarek et al. 2022; Langford et al. 2023).

The China-Indonesia carrageenan relationship is based on strong complementarities. Indonesia has extensive tropical ocean resources populated by low-income farmers that have the knowledge and flexibility to produce seaweed in accordance with tidal patterns, seasonal variations and potential shocks such as extreme weather conditions and pandemics (Mariño et al. 2019; Nuryartono et al. 2020; Langford et al. 2021). The Government of Indonesia has ambitious plans to expand seaweed production to most of the Indonesian provinces (Presidential Regulation No 33,2019). China has strong competitive advantages in manufacturing for export markets or burgeoning domestic markets (Chen et al. 2020). The relationship has been facilitated through favourable trade and investment policies.

The relationship is not however, static. The Chinese carrageenan sector is under pressure, especially from increasing labour costs and environmental standards, prompting outward investment to Indonesia and other countries. While this advances Indonesian objectives to increase domestic “value adding”, there is widespread domestic concern about the potential for foreign companies to outcompete local companies, the high trade dependency on China and the vulnerabilities to capital control or shocks from foreign direct investment (FDI) inflows (Ningrum 2016; Komarek et al. 2022; Saragih et al. 2022; Langford et al. 2023). Despite the growing importance and implications from the rise of China in the global carrageenan industry, there is a dearth of data on the topic from either scholarly or industry sources. A search of extant literature revealed that no other studies have examined the Chinese industry with the exception of a consulting report (IFC 2007). Furthermore, data on the trade and investment linkages with the other key actor in the global industry, Indonesia, is fragmented and highly incomplete. To fill the gap, this paper triangulates a range of data including open-source industry data in multiple languages; statistical data from multiple databases; and interviews with government and industry sources in Indonesia. Given the paucity of data on critical aspects of the global carrageenan seaweed industry, this paper aims to make a contribution primarily through providing a comprehensive, up-to-date and rigorous descriptive analysis. The data is designed to provide an empirical base for further application by industry, policymakers and researchers.

The paper proceeds as follows. The next section introduces data sources and methods used in the analysis. “The Indonesia-China nexus of the global seaweed-to-carrageenan” reviews the evolution and current landscape of the Chinese carrageenan industry, followed by supply chain mapping. “Development of the carrageenan industry in China” discusses major challenges in both seaweed supply and carrageenan demand. “Chinese investment in Indonesian carrageenan processing” raises the implications for both China and Indonesia, especially for Chinese investment in Indonesia. A brief conclusion is presented in “Conclusions”.

## Methods

Given the importance of the Chinese carrageenan industry and the relationship with the Indonesia, literature on the topic is inordinately small, incomplete and outdated. Data are therefore inherently difficult to collect and verify and may be subject to limits in validity and reliability. To build a reliable and robust account of industry developments, we employed a two-step cross-verification process of three types of data: open-source qualitative data, statistical data; and primary data. In the first step, we checked the reliability within each type of data. For example, information on Chinese companies from their websites was verified against credit-checking agencies. Second, we conducted cross-verification between the different types of data. For example, secondary data on Chinese outward investment was checked with primary data collected from Indonesian industry actors and Indonesian export statistics. The triangulation of data independent from each other – that is, collected through separate channels – overcomes problems of dependence upon a single type of data and analysis. Propositions confirmed by two or more independent measurement processes increased the validity and reliability of the conclusions (Webb et al. 2000).

Open-source qualitative data was collected through an exhaustive review of English, Chinese and Indonesian language publications, reports, industry yearbooks, policy documents, company files and media articles. Policy documents in China included quality, application and packaging standards of carrageenan issued by the State Council, the State Administration for Market Regulation and the National Health Commission, and a range of planning documents. Data on Chinese carrageenan manufacturers and distributors was collected from two B2B websites, cross-verified with three company credit checking websites. Further data such as company location, company type, established year, registered capital, processing capacity and product quality certification were collected from individual company websites and, for listed companies, annual reports. The locations of the Chinese carrageenan companies were mapped using ArcGIS 10.7.1.

Statistical analysis was conducted on production statistics from the UN Food and Agriculture Organisation (FAO), Statistics Indonesia (BPS) and China's National Bureau of Statistics (NBS). It is important to note however that the position and volume of Indonesian production was based on FAO (2022) statistics, which are widely argued to be over-stated (Porse and Rudolph 2017; Neish 2020; Hermans 2023; Langford et al. 2023; Rieve 2023) due to issues such as inaccurate assumptions and estimates regarding seaweed cultivation areas, productivity rates and seasonal variations, as well as underreporting of actual output by seaweed processors for tax purposes (Blueyou Consulting Ltd 2016). Indonesia remains an extremely important supplier of carrageenan seaweeds to the global market (Valderrama et al. 2015; Campbell and Hotchkiss 2017; Kambey et al. 2020; Hatch Seaweed Insights 2023a). Trade statistics on the other hand are generally more reliable as they are based on actual trade transactions and are subject to regulations and standardized reporting procedures, making them less susceptible to manipulation or misreporting. Trade statistics were drawn from UN Comtrade (6-digit HS codes) and China Customs (to 8-digit level). This was because the six digits of the Harmonized System (HS) code are universally recognized as a universal code, while additional longer codes may vary across countries for subclassification purposes such as GST rates, declaration process or inspection and quarantine supervision requirements (China Briefing 2020). As a result, even if the product is identical, the succeeding digits of the HS code may not match among countries. For instance, Indonesia and China have assigned different 8-digit HS codes to HS 130239 (mucilages and thickeners), making their codes unable to correspond. Indonesia's 8-digit HS codes include 13,023,911 for SRC, 13,023,912 for RC, 13,023,913 for ATC, and 13,023,919 for other carrageenan, while China's 8-digit HS codes include 13,023,911 for carrageenan, 13,023,912 for alginate, 13,023,919 for other seaweed mucilages and thickeners, and 13,023,990 for other vegetable mucilages and thickeners (China Customs 2023). Therefore, Indonesia's 6-digit HS 130239 matches only with Chinese 8-digit HS 13023911. Amongst other uses, the statistics were used to construct detailed supply chain maps and trade flows in Microsoft Power BI.

In addition to secondary data, the paper also drew on discussions with a range of stakeholders in the Indonesian seaweed industry. This included government officials from the Department of Marine Affairs and Fisheries, seaweed industry associations (Jaringan Sumber Daya (JaSuDa), Indonesia Seaweed Industry Association (ASTRULI) and the Indonesian Seaweed Association (ARLI)) and local seaweed exporters and carrageenan processors interviewed from July 2021 to December 2022. As is the case with other transnational studies (Woodworth et al. 2022), COVID-19 precluded primary fieldwork in China.

Despite our best efforts, one limitation of the study is the inability to fully trace the production and trade of different types and grades of carrageenan into China. Carrageenan can be classified into three major types – kappa, iota and lambda carrageenan, depending on which seaweed it has been extracted from and the number of sulphate groups they contain (Campo et al. 2009; Pereira 2018). Kappa and iota carrageenans are extracted from *Kappaphycus* and *Eucheuma,* respectively, and are the most commonly types used in the industry due to their gelling properties (Bui et al. 2019), accounting for 68% and 24% of the global carrageenan market by volume (Grand View Research 2023). Lambda carrageenan is primarily extracted from wild-harvested *Chondrus crispus* and less commonly used (9%) in the food industry as it does not gel and has low viscosity (Hotchkiss et al. 2016). Additionally, they can also be classified into three distinct grades – alkali treated cottonii (ATC), semi-refined carrageenan (SRC) and refined carrageenan (RC), based on the extraction method used. The processing of ATC involves a series of steps, including washing the seaweed to remove impurities such as sand and salt, treating it with hot alkali to increase its gel strength, and drying it before chopping or pressing it into chips of approximately 1 cm in length (Komarek et al. 2022). These chips can be ground into powder and sold as SRC or subjected to further treatments such as filtration and purification through alcohol precipitation or gel pressing processes to remove residual substances like cellulosic materials, resulting in RC (Komarek et al. 2022). While carrageenan processing is rapidly growing in China, it remains a relatively small segment of the country's overall food industry and there are no specialized agencies or associations in China that collect detailed information on specific market segments, such as different types and grades of carrageenan. Therefore, carrageenan is used in this paper as a generic term to account for the full range of extracted compounds and this study had to rely on aggregated data from industry reports, yearbooks and trade statistics.

## The Indonesia-China nexus of the global seaweed-to-carrageenan industry

**Fig. 1:**
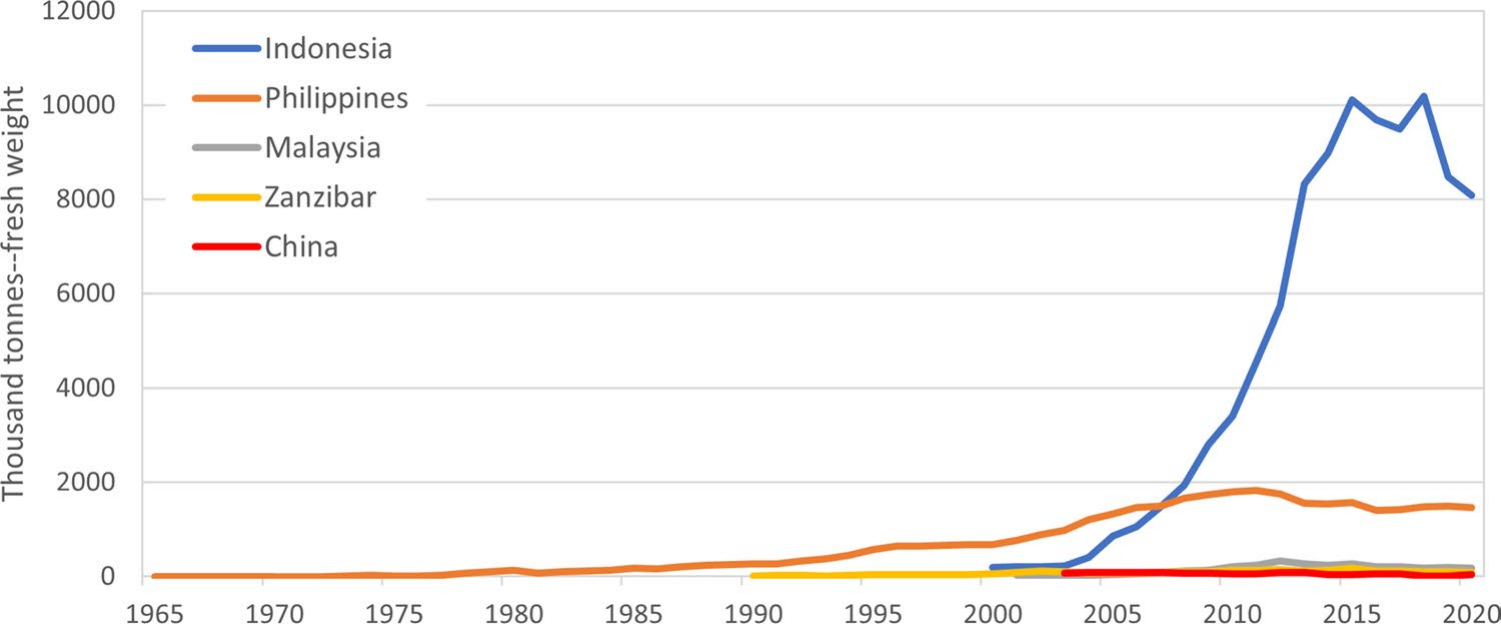
Reported production of carrageenan seaweed in top countries 1965–2020. Source: Data were extracted from the Food and Agriculture Organisation of the United Nations (FAO) database using the FishstatJ software v4.02.07. The carrageenophytes in the FAO database are classified as “Spiny eucheuma” (interpreted as *Eucheuma spinosum*), elkhorn moss (*Kappaphycus alvarezii*) and “*Eucheuma* seaweeds nei” (where nei is “not elsewhere identified”, *Eucheuma* spp). These production volumes are officially published and provide useful long-term indicator of trends but should be regarded as estimates only and are known to be inaccurate. Existing evidence includes Porse and Rudolph (2017), Neish (2020), Hermans (2023), Langford et al. (2023) and Rieve (2023), indicating that Indonesia production volume could be overstated 6–7 times, based on either the reconciliation of statistical sources or according to several industry sources. In addition, the actual carrageenan seaweed production in the Philippines is estimated to be about 5 times lower than the officially reported figures by Blueyou Consulting Ltd (2016) based on declared carrageenan production by seaweed processors. Two factors may explain the discrepancy in reported national seaweed production figures. First, inaccurate assumptions about seaweed cultivation areas, productivity rates and seasonal variations. Second, underreporting of annual production capacity by seaweed processors and carrageenan exporters to evade taxes

While the Indonesia-China relationship has grown to become the most important in the global seaweed-to-carrageenan chain, it has emerged only in the last decade or so. Carrageenan seaweeds were originally harvested wild in north hemisphere but were then cultivated in tropical waters in the Philippines in the 1970s (Neish et al. 2017; Qin 2018) which dominated global production until the 2000s (Fig. 1). Carrageenan seaweed cultivation in the Philippines was accompanied by a wave of investment in processing in the 1990s. Multinational carrageenan manufacturers from United States and Europe (FMC based in USA (was first acquired by DuPont, then merged with International Flavors & Fragrances (IFF) and now operate under the name IFF), Cargill based in USA (through acquiring Degussa(Germany)’s Food Ingredients business, CP Kelco based in Denmark, Kerry based in Ireland and CEAMSA based in Spain) established plants in the Philippines that processed carrageenan both for sale and as one of their raw material inputs (along with others) into their hydrocolloid and food manufacturing lines (Blueyou Consulting Ltd 2016). This was accompanied by the development of domestically owned processing companies (for instance, Shemberg, W Hydrocolloids and TBK). As a result, over 90% of seaweeds from the Philippines is now processed domestically, with minimal quantities exported as raw material (FAO 2022). Furthermore, in response to the inadequate domestic supply, the country has resorted to importing carrageenan seaweeds since 2018 (BFAR 2022). Indonesia has sought to emulate the more integrated Filipino model, with less success so far.

**Fig. 2:**
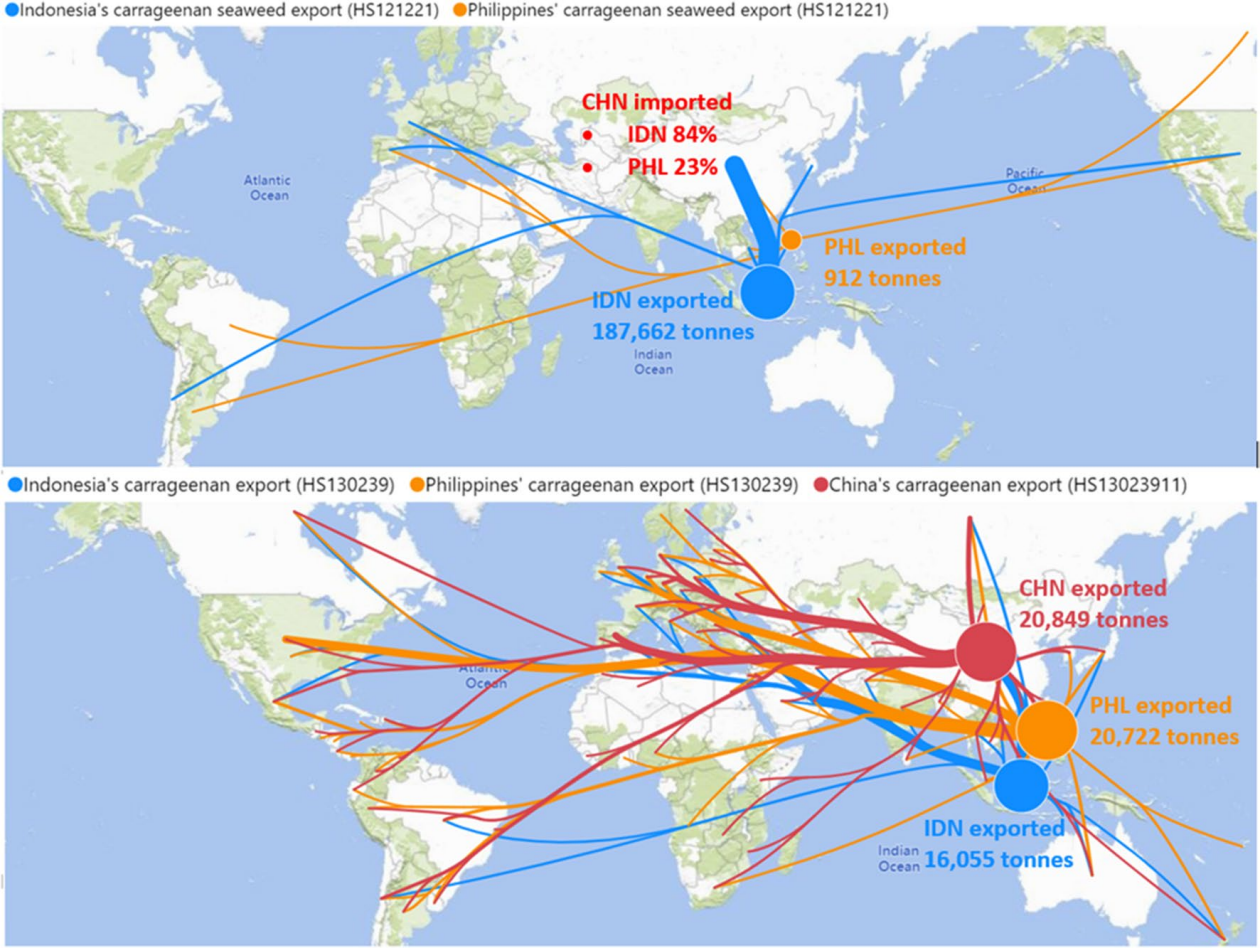
The major global trade networks by export volume in 2021 (**a**) of carrageenan seaweeds and (**b**) carrageenan. Source: UN Comtrade and China Customs, illustrated by the authors using Microsoft Power BI

By 2008, the Philippines lost its dominance in carrageenan seaweed production to Indonesia (Fig. 1) and by 2021 had lost its dominance in carrageenan processing and exports to China (Fig. 2). With disruptions from cyclones in the Philippines and efforts of food companies, processors and scientists sought to diversify and expand production (Ask et al. 2003), carrageenan seaweed cultivation was introduced into Nusa Lembongan in Indonesia in 1987 (Neish et al. 2017). Household-based farming systems expanded into areas like South Sulawesi in eastern Indonesia from 2000 to position the Indonesia as the world’s largest carrageenan seaweed producer. Rapid growth continued until 2015 with a reported annual output of 10.1 million tonnes (fresh weight), but declined from 2018 due to reasons that may include the spread of disease, the decline in strain vigour, environmental pressures, extreme climatic events like heat waves, ocean pollution, labour transition from seaweed cultivation to other sectors like tourism, perturbations in the global carrageenan market, as well as the enhanced statistical reporting (Wiratmini 2018; Media Indonesia 2019; Campbell et al. 2020; Kambey et al. 2020; Ward et al. 2020; Campbell et al. 2022; Duarte et al. 2022). Seaweed cultivation is one of the most economically and socially important aquaculture activities in Indonesia which comprise around 40% of the national fisheries output (Yamin and Tarbuck 2021) and a source of livelihoods for over 62,000 coastal households (BPS 2022 p47).

Unlike the Philippines where virtually all carrageenan seaweeds are processed domestically, approximately 65% of Indonesian carrageenan seaweeds were exported in raw dried form in 2021. This estimate was based on a reconciliation of three statistics series: a) exports of raw dried seaweeds (HS code 121,221); b) thickeners not confined to but dominated by carrageenan (HS code 130,239), which was converted to the RDS through of 4:1 (Komarek et al. 2022); and c) domestic processing statistics of the Ministry of Industry (Kemenperin 2022). Of total exports from Indonesia, 84% was exported to China. The proportional weight of trade flows was indicated in the line (widths and bubble sizes) in Fig. 2 which reported exports volume from the origin countries (Philippines, Indonesia and China) to the destination countries in 2021. China, which was clearly the most attractive market outlet, and commanded the highest prices for most Indonesian seaweed exporters. With 65% of Indonesia's seaweeds currently being exported, this leaves Indonesia 35% for domestic processing. Of the carrageenan produced in Indonesia, approximately 55% was exported. This is shown in the blue line in the lower part of Fig. 2. Of total exports of 16,055 tonnes in 2021, the Ministry of Industry estimated 14% of these exports were in the form of ATC, 58% of SRC and 28% as RC (Kemenperin 2022). In light of the findings by Sudarwati et al. (2020) and Sumule et al. (2021), which reported an 80% proportion of seaweed exports from Indonesia, the observed 65% ratio in 2021 suggests a decreasing proportion in the export of raw dried seaweed. After many years attempting to do so, Indonesia may be gradually increasing proportions processed domestically.

**Fig. 3:**
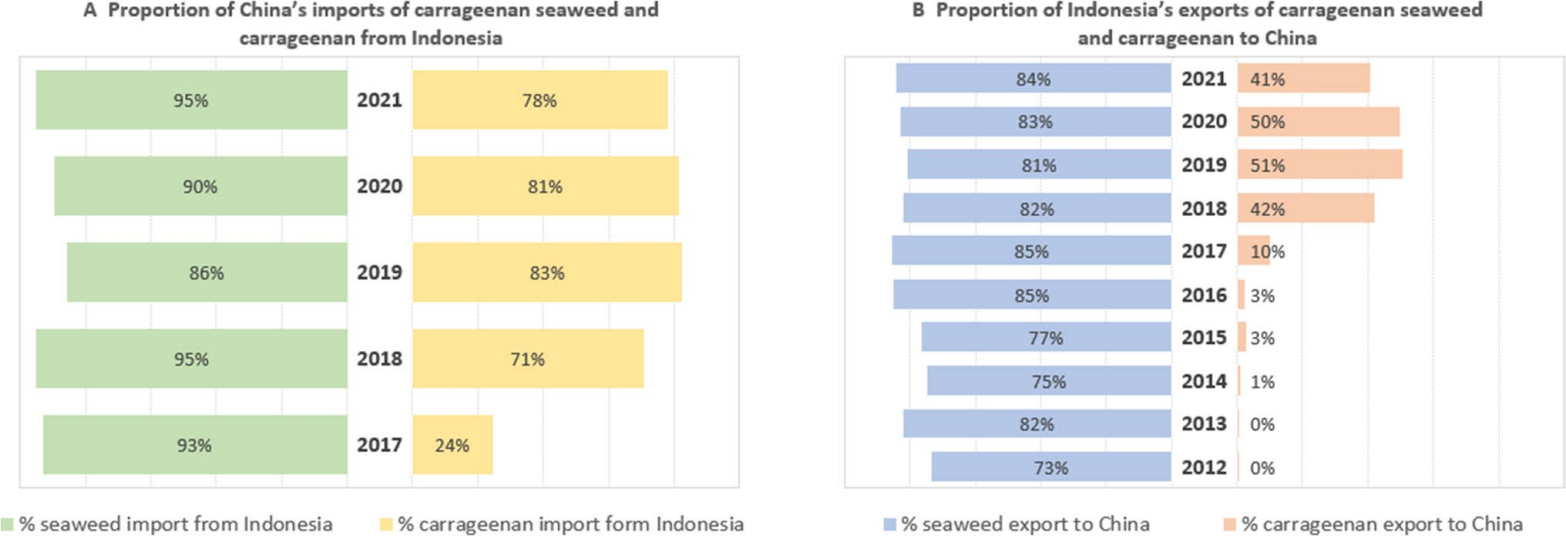
Indonesia and China imports and exports of carrageenan seaweed and carrageenan by volume. Source: UN Comtrade and China Customs

The pivotal role of China as Indonesia's foremost partner in the global seaweed-to-carrageenan industry is a long-standing phenomenon, as demonstrated by the data presented in Fig. 3. China has been the dominant importer of carrageenan seaweed from Indonesia for many years (Fig. 3B). Furthermore, the Indonesian carrageenan industry has experienced a remarkable tenfold surge in both value and volume of exports between 2010 and 2019, with approximately 50% of these exports directed towards China (Fig. 3B). To highlight the interdependence of the Chinese market and carrageenan seaweed trade, it is worth noting that while China plays a crucial role as an important market for many countries, some of these countries’ exports account for only a small fraction of China's imports. However, this is not the case for the trade of carrageenan seaweeds and carrageenan, where Indonesia stands out as a key supplier, with no other country able to match its level of production and volume of exports to China. Figure 3A illustrates that Indonesia has consistently been the primary supplier of carrageenan seaweeds to China for many years, accounting for a significant portion of China's imports (between 90–95%). Specifically, around 80% of China's carrageenan imports are sourced from Indonesia, particularly by Chinese processors that have invested in Indonesia (as detailed in “Chinese investment in Indonesian carrageenan processing”).

**Fig. 4:**
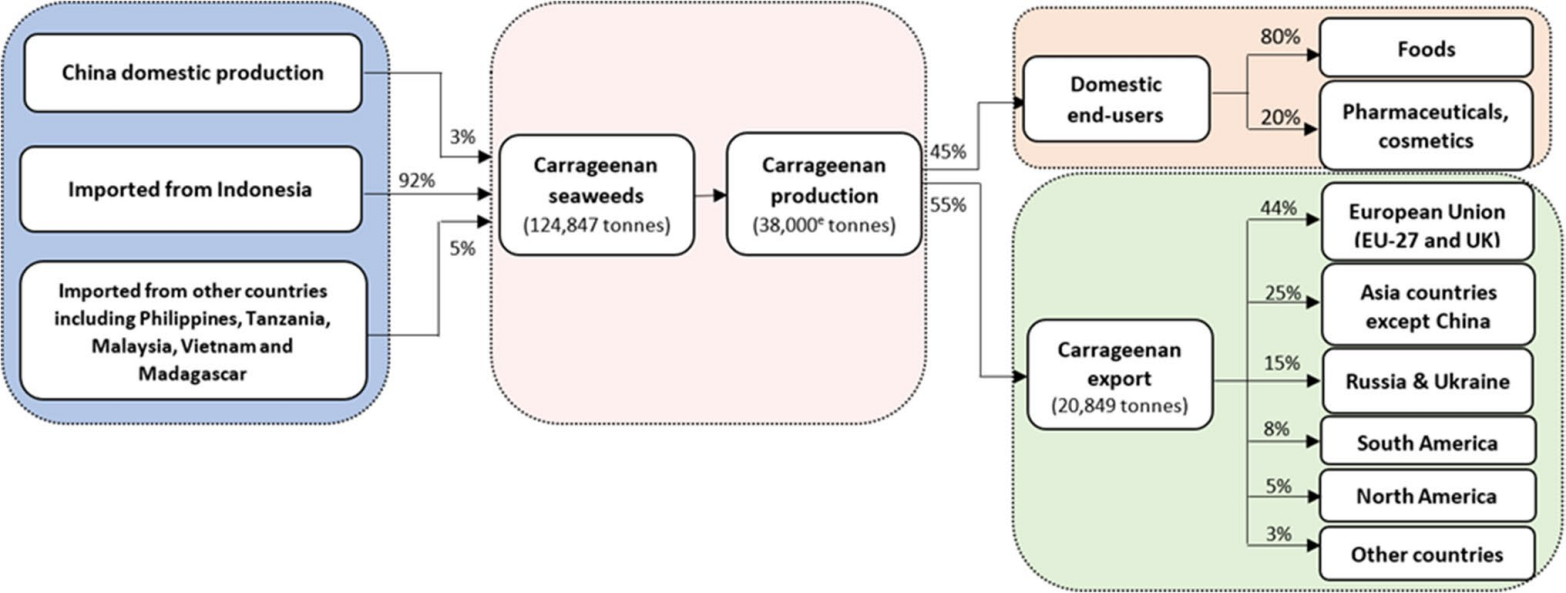
Supply chain of Chinese carrageenan processing, 2021. Data sources: the import volume of dry carrageenan seaweeds and the export volume of carrageenan was collected from China Customs Statistics by 8-digit commodity codes of HS 12122161 and HS 13023911; ^e^ Carrageenan production volume was estimated by authors based on the export ratio to production (Zhang and Duan 2021) and cross-verified by the carrageenan yield ratio from field interview conducted with value chain actors of Indonesian seaweed-to-carrageenan industry in 2022 (Komarek et al. 2022)

China is currently the largest seaweed producer in the world, accounting for 57% of the world’s seaweed production (FAO 2022), although the accuracy of these figures has also been called into question (Hatch Seaweed Insights 2023b). Chinese seaweed production is dominated by *Saccharina japonica* (64%), *Gracilaria* spp*.* (14%), *Neopyropia* spp*.* (8%) and *Undaria pinnatifida* (8%), with other species accounting for 6% according to China’s fishery statistics yearbook in 2020 (ECCFSY 1979–2020). China has in the past tried to develop carrageenan seaweed sector, but this remains small (Fig. 1) due to agro-climatic conditions, disease and competition with other seaweed species (Wang et al. 2020). With rapid growth of the Chinese carrageenan processing sector, the overwhelming majority (97%) of raw material inputs are imported, 92% of which are from Indonesia and the rest from other countries such as the Philippines (3%) (Fig. 4).

Relying on these imported seaweeds, China produces a diverse range of carrageenan through various processing techniques. Of the total, 58% were processed through alkaline extraction to obtain semi-refined carrageenan, while the remaining percentage was processed to refined carrageenan through gel pressing (29%) or alcohol precipitation (13%) (Grand View Research 2023). The resulting semi-refined or refined carrageenan products can be categorized into three types: kappa (67%), iota (24%) and lambda (9%) (Grand View Research 2023), each offering unique functionalities that cater to a wide range of food applications. Nearly half (45%) of Chinese-made carrageenan was sold into the domestic markets, of which 80% goes to the food industry, especially to processed meat, dairy, jelly and soft sweets (Campbell and Hotchkiss 2017). The use of carrageenan in other industries like medicine and biochemistry is not yet widespread. The remaining 55% of Chinese made carrageenan is sold in global markets. China exported 20,849 tonnes of carrageenan in 2021 with a trade value of US$186 million. It exported to 82 countries and regions, but 69 countries imported less than 2% of Chinese exports (< 400 tonnes). Exports are dominated by a handful of regions – 44% to the EU, 25% to other Asia countries and 15% to Russia and Ukraine, with little to the US for several reasons. First, the Philippines has a long-established trade relationship with the US (BFAR 2022). This historical relationship may have contributed to the Philippines' continued export of carrageenan to the US market. Secondly, differences in trade policies may also play a role. The European Union (EU) and China have a trade agreement that allows for lower tariffs (zero tariff for carrageenan import), which may make it more attractive for Chinese exporters to target the European market. This contrast with less favourable trade policies with the US. In the “trade war” between China and the US from 2018, carrageenan was included in the Sect. 301 Tariffs list 4A (US Harmonized Tariff Schedule 9903.88.15). Although the US has agreed to reduce its additional duties from 15% to 7.5% (effective from 14^th^ February 2020), this may still put Chinese exporters at a disadvantage compared to the Philippine and Indonesia exporters.

China's lack of domestic carrageenan seaweed supply may seem like a competitive disadvantage, but this is offset by a range of factors and policies. China has a liberal trade policy on carrageenan seaweeds and carrageenan. The import of carrageenan seaweed (HS 12122161) and carrageenan (HS 13023911) from the 10 ASEAN (Association of Southeast Asian Nations) countries – including Indonesia and Philippines – are subject to China Import Duty for Free Trade Conventional Country of zero (China Customs 2023). This is lower than the 7.5% tariff rate applied to Asia Pacific Trade Agreement Nations (including India and Korea), the 15% tariff rate afforded to Most Favoured Nations (MFN) and 80% to Non-MFN. For Chinese carrageenan exports, the 13% value added tax (VAT) is refundable (China Customs 2023). In addition, Chinese seaweed buyers have, since the early 2010s, been said to use “campaign buying” schemes to lower the cost of their purchases by concentrating purchases within a short period when the price is low and there is a relatively abundant supply of seaweed (Bixler and Porse 2011). Perhaps most importantly, a lack of domestic seaweed supply is offset by the competitiveness of China’s domestic processing sector. In addition to seaweed inputs, chemicals such as alkali solutions are another important input into carrageenan processing, which are domestically sourced. Chinese carrageenan processors are known for their agile approach, high and innovative manufacturing skills, low-cost process chemicals, and skilled work force (Porse and Rudolph 2017).

Due to these alignments, the annual output of Chinese carrageenan increased by 20% in 2018 to 38,000 tonnes in 2021, around one-third of global production (Grand View Research 2023). In addition, Chinese carrageenan companies invested in Indonesia produce perhaps another 10,000 tonnes (see “Chinese investment in Indonesian carrageenan processing” below). The Chinese industry appears well placed to capitalise on global industry growth. The global industry is estimated to processes up to 104,125 tonnes of carrageenan per year and has been forecast to grow at annual compound annual growth rate of 3.8% from 2018 to 2030 (Grand View Research 2023). Drivers include the growth of the processed foods sector and a large range of other applications where carrageenan is blended for it’s natural, colourless, odourless and Halal-conforming characteristics.

**Fig. 5:**
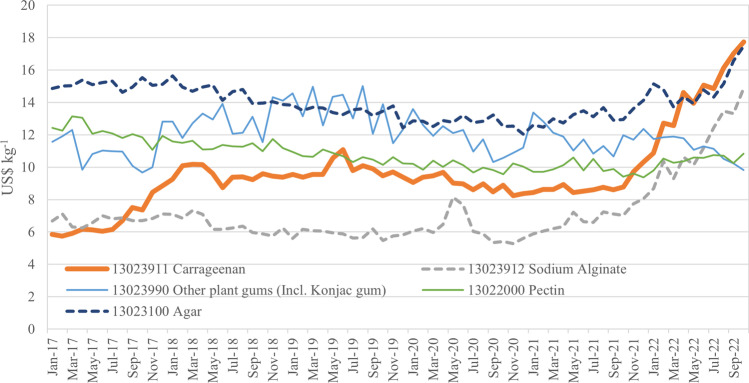
Average unit values for selected gels and thickening agents of Chinese export, 2017–22. Source: China Customs

It is important to note, however, that growth of the Chinese carrageenan industry may not be inevitable or linear. Carrageenan can be substituted with a large number of other gelling agents or thickeners for many applications, depending on the performance of the input relative to price. Based on Chinese export data (Fig. 5), carrageenan prices more than doubled between October 2021 and 2022, to exceed for the first time the price of agar, although prices for other gels (sodium alginate) also increased rapidly. The price data suggests that there has been a general increase in demand for gelling agents, potentially driven by the growing demand for processed foods and changing global economies. However, in current economic conditions of price inflation and economic stagnation (Ha et al. 2022; Ozili and Arun 2023; Weber et al. 2023) places pressure on both consumers and food manufacturers to seek value-for-money inputs and reduce production costs (Campbell and Hotchkiss 2017). Moreover, despite carrageenan's extensive historical usage in the food industry and its compliance with strengthened regulations, it has become the subject of increased regulatory and consumer scrutiny due to concerns regarding its safety (Thomas 1997; Bixler 2017; EFSA et al. 2018). These may lead to an intensification of competition in the application market and provide opportunities for less expensive alternatives (such as Konjac gum) to capture market share.

**Fig. 6:**
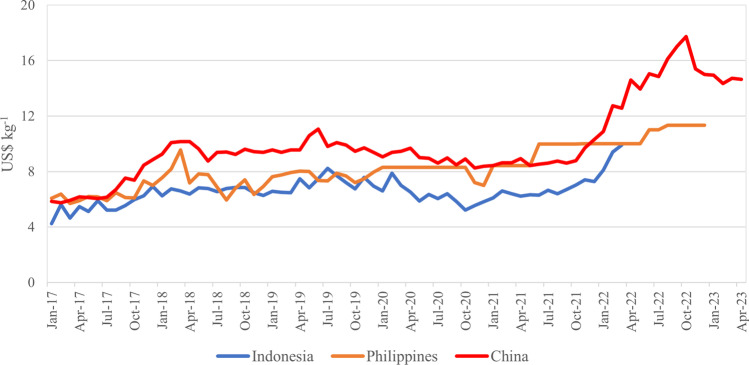
Average monthly unit values of carrageenan exports from selected countries, 2017–23. Source: UN Comtrade and China Customs

Export data also provides insights into the carrageenan processing sector of China in comparison to other counties. Contrary to expectations that China is a low-cost and low-value manufacturer, the average unit value of carrageenan from China was higher than that of the Philippines and Indonesia in most cases between 2017 and 2023 (Fig. 6). This may have been because of rising costs in China (see “Drivers of investment” on drivers of Chinese foreign investment in Indonesia), but also because the types and grades of carrageenan products and qualities varies by country. These factors are not captured in the 6-digit trade statistics of UN Comtrade. In support of the latter case, Chavez et al. (2020) noted that carrageenan produced by China was cheaper than that of those offered by the Philippines and Indonesia if compared by grade during refining. The average unit value of carrageenan from Indonesia was lower than that of the Philippines from 2017 to 2021, which implied that Indonesia carrageenan was price competitive with the Philippines, although this was related to not only product grades but also exchange rates (Chavez et al. 2020).

## Development of the carrageenan industry in China

### The evolution of the Chinese carrageenan industry

**Fig. 7:**
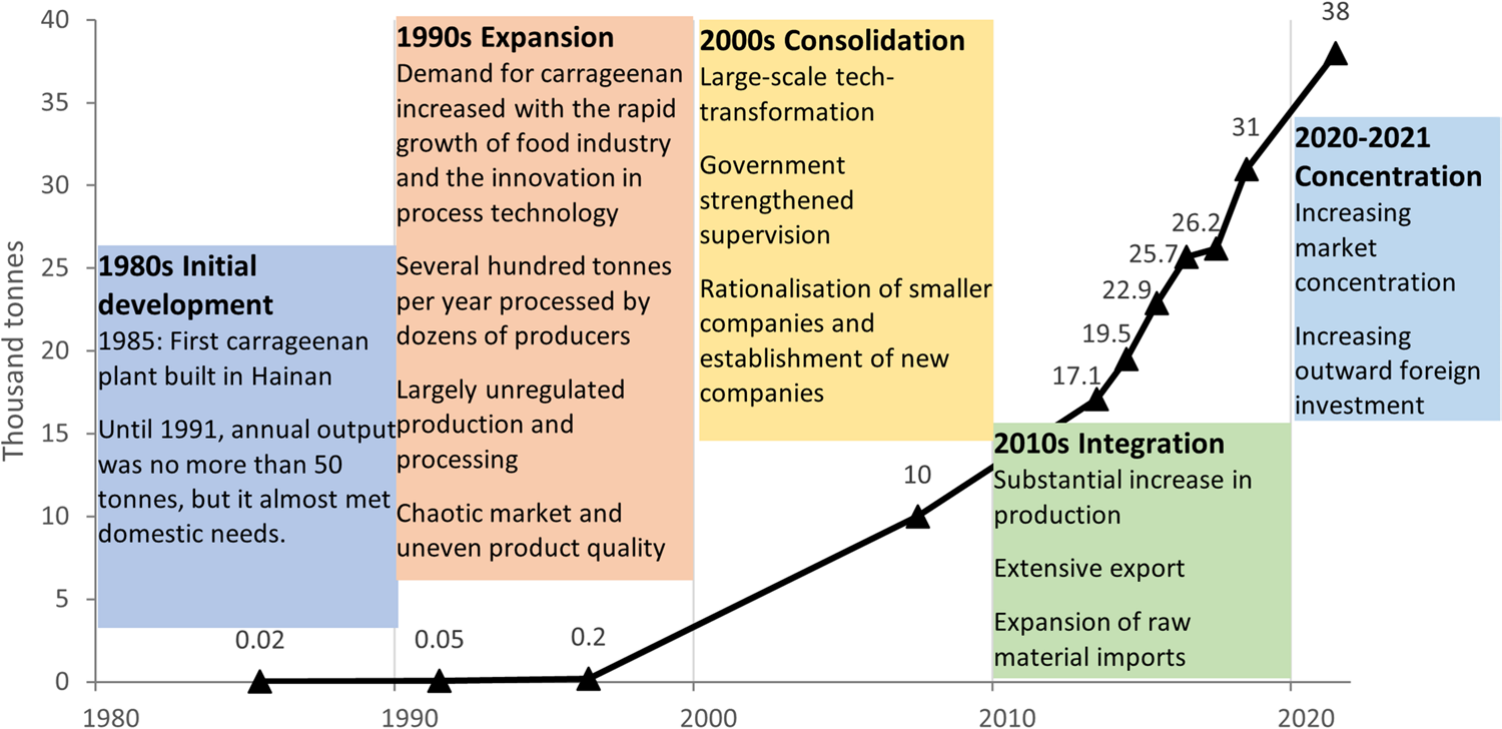
Stages in the development of the Chinese carrageenan industry. Source: Carrageenan production for 1985, 1991, 1996 and 2007 are drawn China Food Industry Yearbooks (ECCFIY 1984–2020). Production data for 2013–2018 are from Zhang and Duan (2021). Qualitative data is drawn from China Fishery Statistical Yearbooks (ECCFSY 1979–2020) reports cited below

This section outlines the extraordinary structural change and growth experienced by the Chinese carrageenan industry through a categorisation of five stages. In the first stage, set in the Central Planning era of the People's Republic of China, food processing and preservation was based on traditional methods, with manual processing or simple equipment and almost no formal food additives industry as recorded in the 1984 China Fishery Statistical Yearbook (ECCFIY 1984–2020). One exception was a state-owned enterprise (SOE) established in 1958, reported to have processed some carrageenan (IFC 2007 p18). Subsequent developments in the post-reform era are summarised in Fig. 7.

The first specialised carrageenan plant recorded in official documents was established in 1985 (ECCFIY 1984–2020). It was located in Wenchang County in the southern, sub-tropical island of Hainan, which was deemed suitable for the cultivation of carrageenan seaweeds. After several research and commercial trials, carrageenan seaweed production in China remained small-scale, so production statistics were not published in national fishery statistical yearbooks until 2003 (ECCFSY 1979–2020). The processing sector had, however, begun its development process. In 1987, carrageenan was reported by the Ministry of Health as one of the thickeners to be included in the hygienic standards for the use of food additives (ECCFIY 1984–2020). The annual output of carrageenan before 1990 was however only in the magnitude of tens of tonnes, sold through a few limited distribution channels with few applications in the food processing (ECCFIY 1984–2020).

In the 1990s expansion phase, more and better thickeners were demanded to fill the increased consumption of processed foods like dairy products and processed meats. Enabled by the development of SRC technology, dozens of enterprises that were not specialised in carrageenan production – but that included carrageenan in processing operations – proliferated across China under various ownership structures, especially collectively-owned township and village enterprises (TVEs) that mushroomed under fiscal decentralization policies that allowed local government to retain locally generated revenues. TVEs in the era were under pressure to be efficient, competitive and profitable within the context of China’s increasingly market oriented economy (Harvie 1999). However, the processers could only source a few hundred tonnes of carrageenan seaweed from within China, and supply from imports was small-scale and erratic in quantity and quality according to the China Food Industry Yearbook in 2000 (ECCFIY 1984–2020). Production was small-scale, fragmented and based on basic equipment and technologies. Regulations were undeveloped since the production was only a few hundred tonnes of uneven quality and not to export standard.

While the 1990s saw a rapid proliferation of small labour-intensive enterprises, the 2000s were a period of technological transformation and enterprise consolidation. Ageing equipment and outdated technology had become one of the main factors restricting the sustainable development of the Chinese carrageenan industry (Guo 2013). This required processers to access higher levels of capital, technology and input supply (Harvie 1999), much of which came through the enterprises that were entirely owned by investors from outside of mainland China, or joint ventures between TVEs located in the coastal region and enterprises based in Hong Kong, Macau and Taiwan. Investors from these regions have been subject to China’s foreign investment laws (NPC 2019), which offered them preferential treatment and less restrictive restrictions than those non-Chinese investors. Linkages with research institutes and universities were also formed (Jin et al. 2017). At the same time, increasing government supervision and rising consumer demand forced food manufacturers to raise food quality and safety standards and the consistency of their raw material inputs as shown in the food industry yearbook of 2008 (ECCFIY 1984–2020). Factories that could not make the advances were forced to exit the industry, replaced by those that could operate in a more rationalised and specialised industry. By the late 2000s, China’s carrageenan output exceeded 10,000 tonnes per year.

In the 2010s, carrageenan production capacity increased further through technological upgrading and closer integration with raw material suppliers. Product quality of carrageenan improved and processors actively explored supply channels in the Philippines and Indonesia. Awareness of food safety increased due to the impact of events such as melamine scandal of the dairy industry, which prompted further strengthening of food safety regulations (Pei et al. 2011). A number of well-known brands and internationally competitive industrial clusters were formed in geographic locations (e.g., the Henan-Shandong and Fujian-Guangdong clusters), through technological initiatives (e.g., marine bio-industry cluster in Shanghai, Shandong and Guangdong) or through the availability with low-cost labour and energy (e.g. Henan Province). The concentration of the domestic carrageenan industry increased, where the output of the top three companies (e.g., Shanghai Brilliant Gum, Green Fresh and Longrun-Newstar) exceeded 60% of the national carrageenan output. By the end of 2018, China’s total carrageenan output exceeded 31,000 tonnes (Zhang and Duan 2021).

### Food safety and environmental regulations

As suggested above, government regulations have had a considerable impact on the development of China’s carrageenan industry, with implications for integration into import and export markets. The carrageenan industry is subject to general laws and regulations pertaining to food additives, which are enforced by three regulatory authorities responsible for its supervision. The first authority is the Ministry of Ecology and Environment (MEE), which was established in March 2018 through the merger of the former Ministry of Environmental Protection and six other government agencies. One of the key tasks of the MEE is to oversee pollution prevention and control, including monitoring the implementation of emission reduction targets and issuing pollutant discharge permits. Additionally, MEE is responsible for reviewing environmental impact assessments for major economic policies and projects pertaining to the carrageenan industry. The second is the State Administration for Market Regulation (SAMR) which replaced the previous State Food and Drug Administration which has jurisdiction over the production and use of food additives, as well as the inspection and quarantine imported and exported food additives. The third is the National Health Commission (NHC) which replaced the previous National Health and Family Planning Committee and formulates national food safety standards for food additives and manage the registration of new food additives.

The carrageenan extraction process involves high usage of alkali, acids and salts and requires water and energy for heating and additional processes to recover and purify carrageenan from seaweed biomass (Chen et al. 2016; Mulyati et al. 2020; Olatunji 2020). Under China's environmental protection legal system, carrageenan manufacturers are required to submit an environmental impact assessment (EIA) report to the MEE during the initial stage of plant construction (Yang et al. 2023). As part of the application-approval process, the plant owner typically commissions a technical contractor to prepare the EIA documents, which must be publicly disclosed for 7–10 days through various channels such as online platforms, newspapers, and announcements to solicit feedback related to the environmental impact of the proposed project. The MEE agency at different levels then reviews the report. The environmental impact reports of several carrageenan processing plants (BLG 2017; Fujian Huanyu Marine Biotechnology 2022), report significant amounts of toxic waste streams from the extraction process, including wastewater, exhaust gas, solid waste, and noise. Wastewater is mainly generated from washing, dehydration, heating, and workshop ground cleaning where the total volume depends on the scale of production. For example, BLG (2017) reported a wastewater volume of around 40.86 tonnes for every tonne of SRC and 70 tonnes for every tonne of RC, while Fujian Huanyu Marine Biotechnology (2022), with an annual output of 500 tonnes of RC, reported over 100 tonnes of wastewater volume for every tonne of RC produced. Some chemicals, such as sulfuric acid and sodium hypochlorite, may enter into the wastewater after use, but the volume is relatively small. The wastewater is typically pre-treated by self-built sewage treatment facilities before being discharged into the collective sewage treatment plant for centralized treatment. Exhaust gas is generated during the crushing and grinding of raw materials (calculated according to 0.1% of the product volume) and from odour gas during alkali treatment and wastewater treatment. Solid waste includes sediment washed from seaweed, filter residue from the filter press, recycled dust, raw material packaging (barrels and bags), and wastewater treatment sludge, which can be used as agricultural crop substrates and soil. Finally, the noise from mechanical operations of production equipment such as pulverisers, colloid mills, and centrifuges is typically recorded at around 80 dB when measured one meter away from the device. Overall, waste disposal has remained a persistent challenge and represents a significant production cost. Therefore, every carrageenan project requires the development of appropriate waste treatment measures and significant investment to address issues such as wastewater, waste gas, noise, and solid waste. To ensure compliance with environmental regulations, measures such as the construction of sewage treatment facilities, installation of dust collectors, anti-seepage and hardening of factory areas and workshop grounds, as well as the construction of general and hazardous waste temporary storage rooms, must be implemented.

In other regulatory procedures, enterprises intending to engage in the production of food additives are also required to obtain a food additive Production Permit and a Pollutant Permit in accordance with the laws “Measures for the Administration of Food Production Licensing” (SAMR 2020) and “Regulations on the Administration of Pollution Discharge Permits” issued by the China State Council (2021). The Measures stipulate that an applicant for a food additive Production Permit shall have the places, production equipment or facilities, food safety management personnel, professional technicians, and management rules suitable for the varieties of food additives that it produces. Government has also introduced regulations on discharging enterprises and strengthened enforcement of pollution discharge permits and penalties for non-compliance. In particular, fines and penalties have been increased up to Rmb 1 million (around US$150,000) for illegal discharge of pollutants. For severe violations, the Regulations impose administrative sanctions, such as production or business restrictions or suspension, or even business shutdown. Moreover, a continuous daily fine system can be levied for repetitive violations or for refusal, or obstruction of inspections. The main responsible person (e.g., director or manager) may face administrative detention or even criminal liabilities for a continuous discharge without a permit after being notified or for the discharge of pollutants by avoiding supervision (e.g., discharge of pollutants through concealed pipes, falsification of monitoring data, abnormal operation of pollutant prevention facilities). In-scope discharging requires entities to record all data and keep it for at least five years. This implication for Chinese carrageenan companies is that regulatory compliance has increased costs.

Regarding food safety, the NHC (2016) announced the National Food Safety Standard for Food additive – Carrageenan (GB 1886.169–2016) which outlines the minimum quality requirements for carrageenan in terms of ash content, lead levels and other criteria. The National Food Safety Standard for the Uses of Food Additives (GB 2760–2014) specifies the basic principles for the application of carrageenan, including its scope of use and allowable maximum dosage. For example, the maximum usage of carrageenan is restricted to 0.3 g L^−1^ for infant formula, 8.0 g L^−1^ for dried noodles, and 5.0 g L^−1^ for maple syrup. Furthermore, the General Rules for Labelling of Food Additives (GB 29924–2013) require that "Carrageenan" be clearly displayed in the prominent place of the ingredients label on food packaging. Food safety regulations are therefore increasingly shaping the global carrageenan market, particularly for high-value applications.

### The contemporary landscape of carrageenan manufacturing in China

**Fig. 8:**
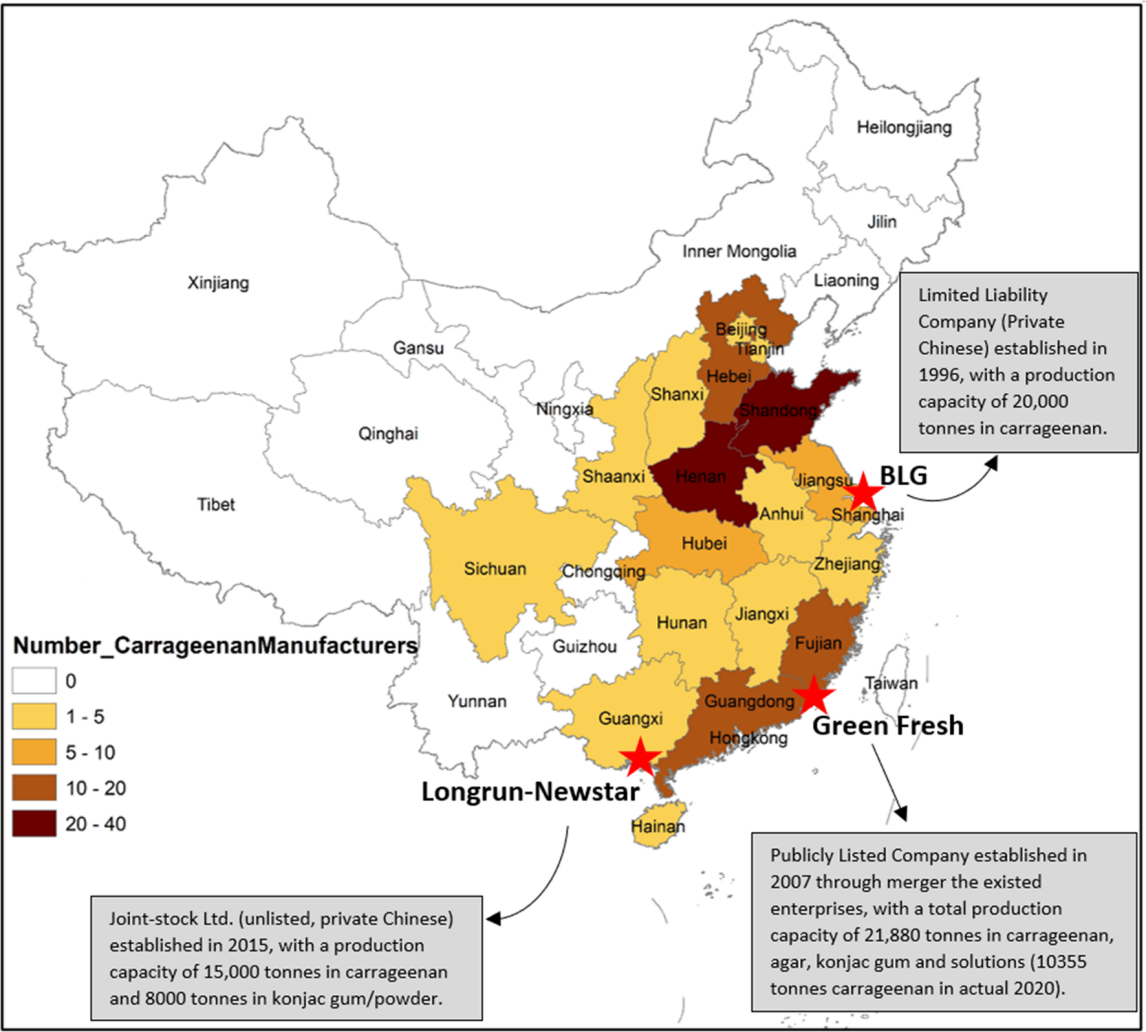
Landscape of current carrageenan manufactures in China. Data source: Chuanglian edible gum network (*chuanglian shiyongjiao wang*, http://www.shiyongjiao.cn/company/011000/; accessed on 24 September 2021) and List of carrageenan companies in China (https://www.listofcompaniesin.com/china/carrageenan/, accessed on 24 September 2021)

The historical evolution of the industry and regulatory settings have given rise to the contemporary Chinese carrageenan industry. By mid-2021 around 150 Chinese companies processed carrageenan, double the 50–60 reported in 2007 by IFC (2007 p12). There are also about 250 companies that do not produce carrageenan, but which distribute or wholesale it. As shown in Fig. 2017,8, carrageenan manufacturers are spread over central and southeast China, with no producers in the remote or less developed northwest and northeast regions. While there were reportedly 20 producers in Hainan (IFC 2007 p13) all but two were closed, mainly due to unsuccessful commercial-scale cultivation of carrageenan seaweeds in the region (Hurtado et al. ), coupled with other reasons such as limited research and development efforts, and the distance from major carrageenan users (IFC 2007 p16). The top three manufacturers (marked by a red star in Fig. 2) are all located along the southeast coastline, with many small-scale carrageenan manufacturers located in Henan and Shandong province which is the transportation centre of China and close to the highest density of food companies. State-owned enterprises have also exited the industry leaving an ownership structure dominated by limited liability companies, invested by private Chinese (natural person) or private Chinese companies (juridical person).

It is not possible to quantify the production volumes of individual companies, except for publicly listed companies like Greenfresh that produce annual financial reports. However, scale of production can be inferred from the reporting of registered capital investment. The majority of companies are relatively small, with only about one quarter (36 out of 150) having a registered capital base larger than Rmb10 million (at an exchange rate of 6.9022 = US$1,448,813). It is also important to note that only a few of the companies are specialised in carrageenans. The vast majority are licenced to produce a range of other gelling products including agar, alginates and konjac gum.

### The broader Chinese food industry

**Fig. 9:**
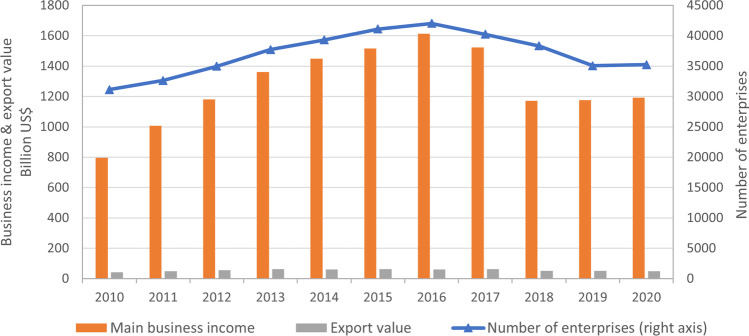
Key indicators in the development of the Chinese food industry. Source: China Food Industry Yearbooks (ECCFIY 1984–2020). Note: The business income data of 2018 cannot be directly compared to the previous year due to changes in statistical collection, where the enterprises that do not meet the industrial statistics requirements (above designated size) was cleared, and the repeated calculation was eliminated in the cross-regional and cross-industry enterprises. Export value was not impacted by the statistical system adjustment in 2018

The prospects of the companies are inextricably tied to that of the broader Chinese food and beverage sector, for which key indicators are shown in Fig. 9. The sector grew significantly from 2010 to 2016, when 42,016 food manufacturing enterprises were above a “designated scale” (*guimo yishang qiye* is a statistical term used in China to refer to industrial enterprises with annual main business revenue of 20 million yuan or more) and generated business income of more than US$1,615 billion at an exchange rate of 6.9022. From a peak in 2016, indictors declined for several reasons: a shift from quantitative expansion to quality improvement; slow population growth (NBSC 2021); and growing demand for safe and higher-quality food (Shi et al. 2011). Exports also declined by 23% due to the US-China trade war that started in 2018 (Bown 2021) and disruptions from COVID (Laborde et al. 2020; Dube et al. 2021). Future trends will change with global economic, trade tensions, technological advances, public health concerns and changing demographics such as population growth, aging populations and changing dietary preferences. These factors are likely to interact in complex ways, and their relative importance may shift over time. As a result, it can be challenging to predict the precise nature and direction of these trends, but it is clear that China's food industry and food exports will need to be adaptable and responsive to changing market conditions in order to remain competitive in the global market.

## Chinese investment in Indonesian carrageenan processing

### Scope of investment

Forces within the domestic and global carrageenan sector have driven large outward investment by Chinese carrageenan companies in Indonesia. This includes major investments by the three largest carrageenan producers in China, BLG, Greenfresh and Longrun-Newstar (Fig. 8). Public information on the investments, and further inquiries with industry associations provide details on the investments are summarised in Table 1 and detailed in “Drivers of investment”.

**Table 1:** Chinese outward foreign direct investment in Indonesian carrageenan processing

Chinese company	Investment company in Indonesia	Year of investment	Location	Ownership	Carrageenan product	Capacity (Tonnes Year^−1^)	Estimated Utilization	Estimated actual production (Tonnes Year^−1^)	Estimated staff numbers
BLG Group (Shanghai Brilliant Gum)	PT Biota Laut Ganggang (BLG)	2015 (operating Oct 2017)	Pinrang Bonded zone, South Sulawesi	Foreign owned (100%)	SRC/RC	8,000	60%	4,800	500 (40 from China)
Greenfresh Food Group	PT Hongxin Algae International	Established in 2012; purchased by Greenfresh in 2021	Surabaya, East Java	Foreign owned (99.83%)	SRC/ATC	3,000 (Capacity planned 7,985 tonnes after acquisition)	60%	1,800	300
Longrun-Newstar Food Group	PT Longrun Carrageenan Indonesia	2016	Kendal Industrial Park, Central Java	Foreign owned (100%)	SRC/RC	3600	50%	1800	300
Fujian Greenone Biotechnology	PT Greeone Biotech	2018	Situbondo, East Java	Foreign owned (100%)	ATC	4,000	60%	2,400	400
Fuyuan Biology Technology	PT Fuyuan Biologi Teknologi	2019	Situbondo, East Java	Unknown	RC	3,000	30%	900	200
Unknown investors from China	PT Bumi Biru Sejahtera (BBS)	2022 (under construction)	Batakte Regency, Kupang	Joint venture	-	-	-	-	-

Sources: information was collected from the websites of the headquarters and branches of each company, cross-verified with JaSuDa in Indonesia

The total processing capacity of Chinese investments in Indonesia is approximately 18,000 tonnes per year. Capacity utilisation of carrageenan manufacturing averages 60% in most plants, which is higher than most Indonesian-owned plants and even those in the Philippines (BFAR 2022), leaving an estimated actual production of 10,000 tonnes for all carrageenan products (ATC, SRC and RC). The Ministry of Industry estimated total domestic production of all carrageenan products as 25,057 tonnes (Kemenperin 2022), suggesting that Chinese-invested plants account for 40% total domestic production. This figure will increase to 60% if further planned investment from Greenfresh actualise. Under-utilised capacity may be the result of insufficient seaweeds supply (BFAR 2022), which limits the ability of manufacturers to operate at full capacity. Nevertheless, this can be advantageous for maintaining pricing power in the marketing and distribution of carrageenan, probably one of the reasons for the price increase in Fig. 5). On the other hand, the company’s decision to further expanding the production capacity may indicate its positive outlook for the future of the industry, since a larger capacity allows them to increase production without incurring expensive overhead costs associated with acquiring new equipment or property (Ge and Ding 2008). Furthermore, the ability to meet unexpected surges in demand or to accommodate potential growth opportunities could also be a significant factor in the decision to establish overcapacity (Dong and Sun 2022).

**Shanghai Brilliant Gum (BLG)** was founded in 1996 and has developed into a major manufacturer and service provider of carrageenan, konjac gum, agar and blended products. BLG has three plants in China – Shanghai, Zhejiang and Ningxia – and one plant in Indonesia. The plant in Ningxia founded in 2021 claimed to be the world’s largest production base covering a 33 ha site for biogums including curdlan, gellan gum and xanthan gum (BLG website 2022). Ningxia is a relatively remote and undeveloped province of China, so may offer investment benefits including lower cost land and electricity, labour and favourable policies and environmental regulations (China Economic Net 2015).

BLG had been scoping investment in Indonesia for many years, and registered investment in PT Biota Laut Ganggang (BLG) in 2015 through an investment vehicle in Singapore (to be eligible for preferential ASEAN investment treatment). The greenfield plant began operations in October 2017 with a daily production capacity of 100 tonnes dried carrageenan seaweeds (although operating at 60% capacity). It is located in Pinrang in South Sulawesi Province, the most intensive seaweed production area of Indonesia. The company sources seaweeds throughout Indonesia but especially South Sulawesi, North Kalimantan and Eastern provinces. It buys through company buyers and trade networks that include seven former seaweed exporters. These are CV Jala Ganggang Bersama, PT Sindo Serene International, PT Mega Citra Karya, PT Rika Rayhan Mandiri, CV Mitra Sejahtera, PT Central Pulau Laut and CV Guna Bahari (JaSuDa 2022). The company has a list of specifications (e.g. moisture: 36 – 38%, dirt 3 – 5%, yield or carrageenan content minimum 25%, light colour) linked to a pricing schedule, and purchase prices are adjusted, often in short term (weekly) basis. BLG Indonesia also sources konjac for konjac gum and *gracilaria* for agar. It uses the Makassar Port to import chemical and other inputs from China and to export ATC and SRC for further processing especially in China, but also the US, EU, Japan and Australia. The company was built predominantly with Chinese labour, and reportedly currently employs approximately 500 staff, of which 40 are from China. The majority of Indonesian workers are from Suppa Sub-district in Pinrang.

**Greenfresh** is the second largest hydrocolloids producer in China, based in Fujian on the east coast. In Indonesia, it invested in a modest scale in establishing Hongxin Algae International in 2012. The initial investment was aimed at securing a stable supply of seaweeds from Indonesia, which is a key ingredient in many of the company's products. By investing in Hongxin Algae International, the company may have sought to establish a strategic foothold in the carrageenan industry and gain greater control over its supply chain. However, the precise motivations and strategic implications of the investment are likely to be multifaceted and context-dependent and may have been influenced by a variety of internal and external factors including market conditions, regulatory frameworks, and competition dynamics. In recent years, Greenfresh reportedly increased its investment in Hongxin through a publicly listed, limited liability company incorporated in Hong Kong, Green Future, reportedly to minimise borrowing costs (Fung 2022). This strategic move has allowed Greenfresh to access new sources of funding and leverage its existing assets and capital. To March 2021, Green Future purchased 99.83% of the Indonesian company with a capacity to process 4,300 tonnes of SRC per year with 60% utilization, and the company plans to increase processing capacity to 10,000 tonnes per year after full acquisition. Greenfresh in Indonesia buys seaweed from East Java, Bali, East Nusa Tenggara and Maluku through local traders. Hongxin exports directly to the Green Fresh plant in Fujian.

**Longrun Newstar** is the third largest hydrocolloid producer in China and produces konjac gum and carrageenans, with a design capacity of 15,000 tonnes of carrageenan per year in China (Longrun Newstar 2023). The Chinese company has various international investments including in PT Longrun Carrageenan Indonesia in 2016, located in Kendal Industrial Park in Central Java. Longrun Newstar has also invested in a konjac gum plant PT Newstar Konjak Nusantar, which appears to be held up (Lantern Today 2022).

The predecessor of Fujian **Greenone Biotechnology** is Shishi Guangyi Foodstuff Co., Ltd which founded production lines in China in 1998. In order to obtain high-quality seaweed, the company claims to have dispatched dedicated personnel to Indonesia and the Philippines all year-round to procure seaweeds (Greenone Biotechnology 2023). Greenone expanded its processing to Indonesia in 2018 to solve the demand for raw material supply. Publicly available details on PT Fuyuan Biologi Teknologi and PT Bumi Biru Sejahtera (BBS) are sparse as they are private enterprises with relatively small-scale or new investments.

### Drivers of investment

The large investments are driven by a range of forces and incentives. These are summarised in an interview with the CFO of Greenfresh (Fung 2022) and analysed further below. The most important driver of the FDI is to capture seaweed supply. As established in “The Indonesia-China nexus of the global seaweed-to-carrageenan industry”, Chinese processors are reliant on importing raw dried seaweeds, which accounts for 70–80% of all costs (BFAR 2022). Chinese processors are therefore sensitive to potential supply disruptions that can come from various sources. In 2011 the Indonesian Ministry of Marine Affairs and Fisheries (Kementerian Kelautan dan Perikanan, KKP) announced export restrictions or taxies on raw dried seaweeds in an attempt to promote the development of the domestic processing sector. While this was not implemented, there were additional discussions of export tariffs and quotas in 2015 (Harrison-Dunn 2015; Wright 2017; Waters et al. 2019). These were accompanied by a period of sharp increases and fluctuations in prices that made it difficult for processors or exporters to enter into long-term contracts. Further disruptions were caused by COVID-19.

While Chinese companies can take measures to counteract possible supply shortages – by stockpiling of raw materials and entering into long-term supply contracts and expanding international sources (Keohane 2016) – investment in Indonesia is a preferred method to reduce the risks of trade disruptions for companies with the capacities to do so. The importance of seaweed inputs in processing means that any measures to reduce costs or maintain physical stability and consistency, including colour, viscosity, gel strength, moisture content, ash content, and particle size distribution, represent a significant source of competitive advantage (Utomo and Mulia 2013). Location close to raw material sources increases knowledge of markets and supply, helps build procurement networks to local levels, potentially reduces transaction and logistics costs and increased market influence through price setting (Kokko 2006; Bi et al. 2020). As a reflection of this, many Chinese companies claim on their websites that they have established reliable seaweed suppliers in Indonesia, including Zhenpai Hydrocolloids and Global Ocean Biotechnology.

Another driver of outward FDI is rising costs in China. One such cost is labour. Data from the Ministry of Human Resources and Social Security of the People's Republic of China shows that the monthly minimum wage across China ranges from Rmb1,550 to Rmb2,480, with an average of Rmb1,824 in 2020 (at an exchange rate of 6.9022 = US$3,171 per year). However, wages in areas where most Chinese carrageenan plants are located (i.e., in eastern and central China) are higher than national average. In comparison, the minimum wage in South Sulawesi Province in 2020 was Rp2,860,383 per month (at an exchange rate of 14,481 = US$2,370 per year), 25% lower than the Chinese national average (BPS 2022 p138).

Second, carrageenan processing is water and energy intensive (Shi et al. 2022) and profitability is sensitive to the costs of electricity, water and steam which are typically supplied by public utilities and charged based on usage (Stanley 1987; Mulyati et al. 2020). These costs have risen significantly in China in recent years, partly due to the 2016 dual-control policy which was to reduce energy intensity and to limit total energy consumption as a key measure that the Chinese government implements to help meet its energy and climate goals. For example, the unit cost of industrial steam increased from Rmb140 tonne^−1^ (at an exchange rate of 6.9022 = US$20.3) to Rmb280 tonne^−1^ (at an exchange rate of 6.9022 = US$40.6) (BLG News 2021). In addition, indiscriminate power cuts and production restrictions – caused partly by local bureaucrats to achieve energy intensity targets – further discourage production. Indonesian industrial electricity prices are 20% lower than China where China is US$0.025 MJ^−1^ and Indonesia is US$0.020 MJ^−1^ (Global petrol prices 2022).

In addition, the Chinese government also strengthened environmental regulations (as discussed in 4.2), which further increased costs in plant investments and management systems. For instance, the treatment and disposal costs of wastewater from processing are Rmb10-11 tonne^−1^ (US$1.6 tonne^−1^). Indonesia has environmental regulations from national to local levels on wastewater treatment and solid waste management, but these are reported to be more relaxed than China (Fung 2022).

The outward investment is also facilitated by favourable policy settings from both China and Indonesia. On the Chinese side, technology development and carrageenan production are listed as an “encouraged” industry under the item of *Agroforestry – food additives* in the Catalogue for Guiding Industry Restructuring (NDRC 2019). This catalogue is considered an important basis for guiding the direction of domestic and foreign investment and guiding government agencies to manage local investment projects and formulate fiscal, tax, credit, land, import and export policies, including the duty-free import of high-tech equipment. Chinese outward FDI is also encouraged under the 2001 “Go Out” policy and the subsequent “Belt and Road Initiative” (BRI) program, including in Indonesia which occupies a central place on its BRI map. The institutional support from the central government enables Chinese enterprises to obtain preferential policies such as loans, capital subsidies and lower investment thresholds, which can help them reduce transaction costs and overcome the liabilities of treatment as foreigners along the BRI route (Yao et al. 2022).

On the Indonesian side, one plant (BLG) is located in a bonded zone (Pinrang) and therefore eligible for preferential treatment including exemption from import tariffs, value-added tax and excise duties, as well as simplification in the import of inputs like machinery and chemicals. There are similar inducements for Longrun-Newstar to locate in the Kendal Industrial Park in Central Java. More generally, Indonesia’s ambition to become the “Global Maritime Fulcrum” aligns with China’s BRI program, which has both direct and indirect implications for investment.

Another feature of the Indonesian seaweed industry is that ethnic Chinese Indonesians dominate post-production sectors. Chinese Indonesians have traditionally played a large role in seaweed trading and exports and own virtually all domestic Indonesian carrageenan processors. For example, Chinese Indonesians have established seaweed processing plants in West Java (Gumindo Perkasa Industri, Galic Artabahari, Hydrocolloid Indonesia), East Java (Algalindo Perdana – Seatech Carrageenan, Amarta Carrageenan Indonesia) and South Sulawesi (Cahaya Cemerlang, Giwang Citra Laut, Wahyu Putra Bimasakti, Anugerah Mapan Jaya Hydrocolloids) (JaSuDa 2022). This follows structures in agriculture-based trade established by early Chinese diasporas (Skinner 1963), that have been observed in the contemporary local-level business activities (Chiang and Cheng 2017) and in fisheries (Novaczek et al. 2001). Large bodies of literature document ethnic Chinese business networks that form alliances with elites and expedite business (McVey 1992) that extend to mainland China through trade and investment flows (Ren and Liu 2022). While market forces drive the growth of particular industries, Chinese Indonesians may have shared cultural and social networks that facilitate business activities.

### Implications for Indonesia

Drivers described above make Indonesia an attractive destination for Chinese FDI and reinforce powerful trade flows. This involves a series of investments that entail both positive and negative implications for Indonesia. On the positive side, foreign investment can promote industry development and generate economic revenues, employment and technology transfer (Denisia 2010). The counter-argument is that inward FDI flows from China reflect the outsourcing tendencies of global value chains to take advantage of Indonesia’s low wages and less stringent environmental regulation, with little potential for technology transfer or industrial upgrading (Wilkinson 2008). These issues are explored in the cases of BLG and GreenFresh.

**Fig. 10:**
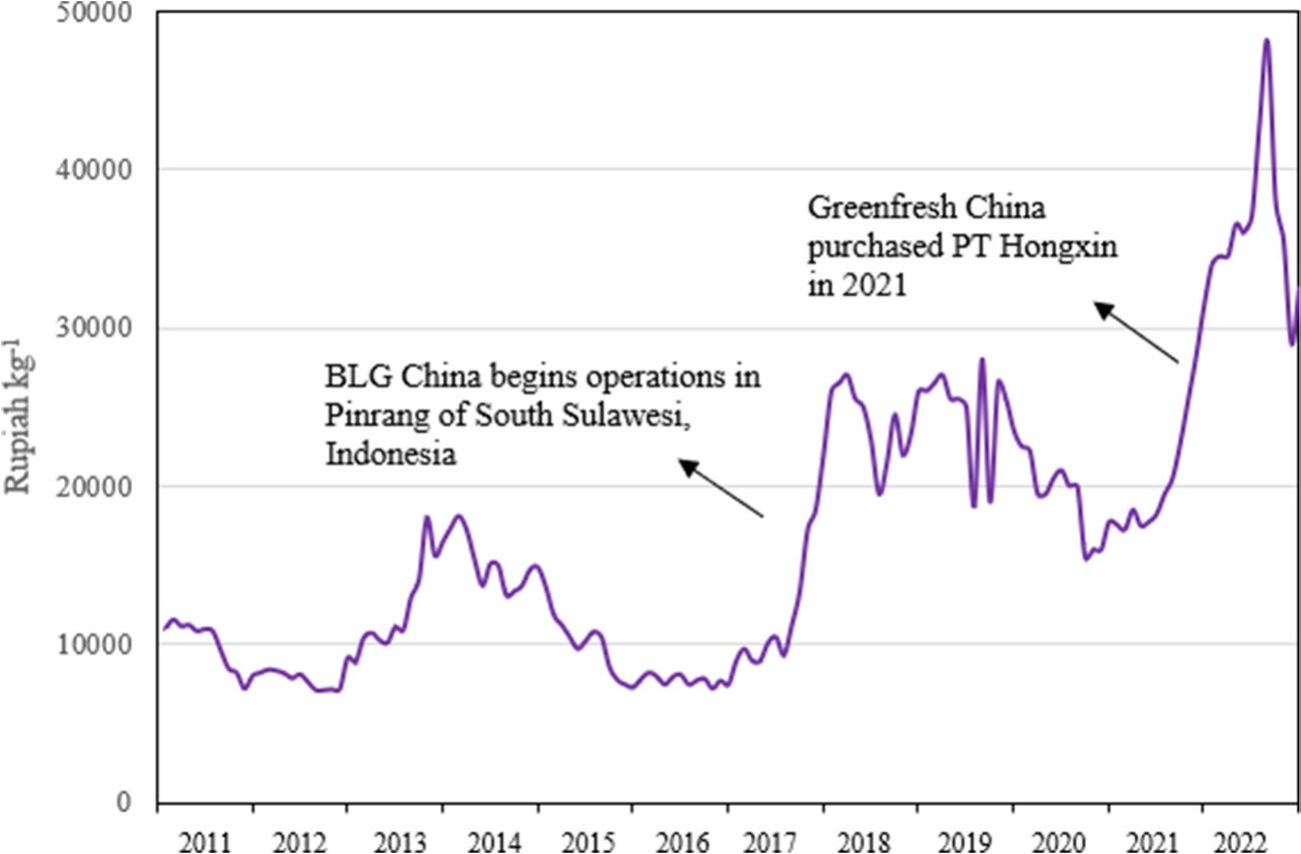
Seaweed prices in Makassar, South Sulawesi, 2011 – 2022. Source: JaSuDa internal data

As mentioned, BLG and Greenfresh employ significant numbers of workers in a labour-intensive activity with a high proportion for the local workforce. BLG has favourable treatment for a range of taxes, including customs duty (for imports and exports), VAT, luxury tax and income tax (reportedly in accordance with Ministry of Finance Regulation PMK 65–2021). They are not obliged to engage in technology transfer, especially as they do not have a domestic joint venture partner. Importantly, Chinese investments are likely to put upward pressure on the prices of various seaweeds that can benefit producers (Langford et al. 2022). This is inferred in Fig. 10 where the operations of BLG in 2017 and the acquisition of Greenfresh in 2021 coincide with two major price spikes in Indonesia.

It is plausible that companies of the scale of BLG and Greenfresh would have a significant short-term impact on prices, especially in early stages as they seek to build inventories to begin operations. Industry actors report that the major Chinese companies – BLG and Greenfresh – “set” prices at the beginning of the week that guide market prices for other actors for the rest of the week. In principle, the FDI can be expected to add to aggregate demand and to diversify sales channels, including sales options for sellers which are for both export of dried seaweed (which remains the dominant channel) and for local processing (both domestic companies and foreign invested). Importantly, there is also intense competition for supply between the two Chinese-invested processors.

While competition and price pressure may be beneficial for Indonesian seaweed producers, it places pressure on domestic Indonesian processors. High prices require high levels of working capital for procurement that may not be available. Upward-trending input prices can cause severe losses if not matched with equivalent increases in output prices set in contracts. Local companies are known to stop processing when price levels get above a point or to switch into the processing of cheaper seaweed. Peak industry bodies voice concerns about the scale and effect of the Chinese investments. They also advocate for equivalent tax treatment – such as duty-free imports of chemicals – so far to no avail.

FDI can also have environmental effects. For example, BLG faced community concerns and protests in Pinrang over effluent from wastewater, argued to cause damage to shrimp farming in violation to Pinrang District Regulation Number 2 – 2016 (Media Indonesia 2022). Insofar as Chinese investment is motivated by increasing environmental standards and costs in China, FDI can be seen as facilitating the “export” of pollution to Indonesia which has lower standards (Fung 2022). As Tritto (2021) highlighted, Indonesia's loose requirements and enforcement on technological standards and environment protection drivers for incoming FDI. While this may hinder technology transfer and the sustainability of the carrageenan processing industry, this may be offset in a scenario where Chinese-invested processors, with more modern wastewater management systems, displace domestic processing with lower-level systems.

## Conclusions

The Chinese carrageenan processing sector has, from a low base, developed to become perhaps the world’s largest, with widespread implications for the broader global carrageenan seaweed industry. The implications are especially direct for Indonesia, which is inextricably bound to China through both trade and investment flows. While this paper seeks to provide much-needed empirical information on the relationship for industry, government and researchers. Several concluding themes are raised that will be fascinating to observe unfold into the future.

The growth of the Chinese carrageenan processing sector has broken the traditional pre-eminence of the US, the EU and the Philippines in the sector. The China-Indonesia nexus can be seen as a parallel and competing chain, to form a somewhat bifurcated global carrageenan value chain. The carrageenan industry provides just one example of the assertion of Chinese manufacturing competitiveness to take up a key central role in global value chains.

On the other hand, carrageenan processing is a highly competitive, low-margin manufacturing sector subject to substantial risks. The “driving point” of the seaweed-to-carrageenan chain – where more value is added – may be further downstream in the blending sector. Solutions providers source carrageenan of various types and other hydrocolloids under contract – increasingly from Chinese companies – to formulate mixed/compound gels for specific food products such as jelly, dairy and meat (Hilliou 2014; Porse and Rudolph 2017). Combinational use of hydrocolloids will be increasingly required to meet future consumer demands, in close coordination with customers (e.g., processed food companies), accompanied by high levels of the research and development (R&D) and technological improvements (Yemenicioğlu et al. 2020).

“Western” countries maintain central position in these downstream sectors at least for “Western” food markets. The question is whether Chinese companies can develop the capacities to take market share in this sector to form the next (sixth) stage in industry development (Fig. 6), not only for the large and burgeoning Chinese food sector, but also for the international and “Western” food industry and consumers. Advances in Chinese food science and investment into high-tech manufacturing suggest so, but there are also managerial, institutional and cultural barriers.

For Indonesia, China offers a market for products in a range of forms – for export as raw dried seaweed, for Chinese companies invested in Indonesia, and for carrageenan in different forms. Thus, Chinese demand for Indonesian seaweeds is not homogeneous, but a series of differentiated channels that compete with each other to provide market options, flexibility and competition. FDI also advances the objectives of the Indonesian government to increase domestic production. With this path now well-trodden by Chinese companies, Indonesian government agencies, at all levels, may wish to impose tax, technology transfer and environmental terms that more directly benefit Indonesia.

## Data Availability

All data analysed during this study are included in the manuscript, further inquiries can be directed to the corresponding author.

## References

[CR1] Ali MKM, Fudholi A, Sulaiman J, Muthuvalu MS, Ruslan MH, Yasir SM, Hurtado AQ, Hurtado AQ, Critchley AT, Neish I (2017). Post-harvest handling of eucheumatoid seaweeds. Tropical Seaweed Farming Trends, Problems and Opportunities: Focus on *Kappaphycus* and *Eucheuma* of Commerce.

[CR2] Ask EI, Batibasaga A, Zertuche-Gonzalez JA, De San M (2003) Three decades of *Kappaphycus alvarezii* (Rhodophyta) introduction to non-endemic locations. In: Chapman ARO, Andrson RJ, Vreeland VJ, Davison IR (eds) Seventeenth International Seaweed Symposium. Oxford University Press, Oxford, pp 49–57

[CR3] BFAR (Bureau of Fisheries and Aquatic Resources) (2022) Philippine Seaweed Industry Roadmap 2022–2026. http://www.pcaf.da.gov.ph/wp-content/uploads/2022/06/Philippine-Seaweed-Industry-Roadmap-2022-2026.pdf. Accessed 31 Jan 2023

[CR4] Bi Y, Ren Z, Bao K (2020). Does distance matter in foreign direct investment sub-national location choice? Evidence from China. Front Bus Res China.

[CR5] Bixler HJ (2017). The carrageenan controversy. J Appl Phycol.

[CR6] Bixler HJ, Porse H (2011). A decade of change in the seaweed hydrocolloids industry. J Appl Phycol.

[CR7] Blanchetti-Revelli L (1997). Keeping meat and dairy consumers slim: Philippine seaweed, American carrageenan and the USFDA. Anthropol Today.

[CR8] BLG (2017) Announcement of environmental impact assessment report: annual production of 2,000 tonnes of IOTA refined carrageenan and 2,000 tonnes of purified konjac gum project EIA report. https://jz.docin.com/p-1957943243.html; accessed 10 May 2023

[CR9] BLG News (2021) Zhejiang factory held a "lean production, eliminate waste” on-site lecture. https://www.sh-blg.com/news/62.html; accessed 31 Jan 2023

[CR10] BLG website (2022) BLG Product Category. https://en.sh-blg.com/Products_list2.html; accessed 27 January 2023

[CR11] Blueyou Consulting Ltd (2016) Business model and financial plan seaweed farming productivity and value chain improvement impact investment for a business venture for community-based seaweed farming in northern Palawan, Philippines, PEMSEA and BLUEYOU Consulting LTD. Zürich, Switzerland. http://seaknowledgebank.net/sites/default/files/Business%20Concept%20Community-based%20Seaweed%20Farming%20Philippines%20-%20FINAL_0.pdf; accessed 10 May 2023

[CR12] Bown CP (2021) US-China Trade War Tariffs: An Up-to-Date Chart. https://www.piie.com/research/piie-charts/us-china-trade-war-tariffs-date-chart; accessed 10 May 2023

[CR13] BPS (2022) Hasil Survei Komoditas Perikanan Potensi Rumput Laut 2021 Seri - 2. Badan Pusat Statistic. https://www.bps.go.id/publication/2022/08/29/269de33babc6e3d52bbae5b6/hasil-survei-komoditas-perikanan-potensi-rumput-laut-2021-seri-2.html; accessed 10 May 2023

[CR14] Bui VT, Nguyen BT, Nicolai T, Renou F (2019) Mixed iota and kappa carrageenan gels in the presence of both calcium and potassium ions. Carbohydr Polym 223:115107 10.1016/j.carbpol.2019.115107 31426987

[CR15] Campbell I, Kambey CS, Mateo JP, Rusekwa SB, Hurtado AQ, Msuya FE, Stentiford GD, Cottier-Cook EJ (2020). Biosecurity policy and legislation for the global seaweed aquaculture industry. J Appl Phycol.

[CR16] Campbell I, Mateo J, Rusekwa SB, Kambey CS, Hurtado A, Msuya FE, Cottier-Cook EJ (2022) An international evaluation of biosecurity management capacity in the seaweed aquaculture industry. J Environ Manage 304:114112 10.1016/j.jenvman.2021.114112 34923419

[CR17] Campbell R, Hotchkiss S (2017) Carrageenan industry market overview. In: Hurtado A, Critchley A, Neish I (eds) Tropical Seaweed Farming Trends, Problems and Opportunities: Focus on *Kappaphycus* and *Eucheuma* of Commerce. Springer, Cham, pp 193–205

[CR18] Campo VL, Kawano DF, da Silva Jr DB, Carvalho I (2009). Carrageenans: Biological properties, chemical modifications and structural analysis–A review. Carbohydr Polym.

[CR19] Chan G, Lee PK, Chan LH (2011) China engages global governance: a new world order in the making? Routledge, Oxford

[CR20] Chavez SANE, Bathan BM, Lizardo RCM (2020). Export competitiveness analysis of Philippine carrageenan products. J Asia Trade Bus.

[CR21] Chen J, Chen D, Yao A (2020). Trade development between China and countries along the Belt and Road: A spatial econometric analysis based on trade competitiveness and complementarity. Pac Econ Rev.

[CR22] Chen S, Ni H, Hao G, Zhuang P, Wang X (2016) Present status of carrageenan industry in Fujian Province. Farm Prod Process 1:53–55

[CR23] Chiang B, Cheng JS, Santasombat Y (2017). Ethnic Chinese Enterprises in Indonesia: A Case Study of West Kalimantan. Chinese Capitalism in Southeast Asia: Cultures and Practices.

[CR24] China Briefing (2020) HS Codes in China – What Should Foreign Investors, Trading Entities Pay Attention To? https://www.china-briefing.com/news/hs-codes-in-china-what-should-foreign-investors-trading-entities-pay-attention-to/; accessed 12 May 2023

[CR25] China Customs (2023) Import and export commodity tax rate query. http://online.customs.gov.cn/ociswebserver/pages/jckspsl/index.html; accessed 8 May 2023

[CR26] China Economic Net (2015) Peng Jianxun, a Taiwanese businessman in Ningxia: five reasons to invest in Ningxia (Zaining taishang Peng Jianxun: Touzi Ningxia de Wuda Liyou). http://district.ce.cn/newarea/roll/201503/31/t20150331_4987556.shtml; accessed 27 January 2023

[CR27] China State Council (CSC) (2021) Regulations on the Administration of Permitting of Pollutant Discharges. Order No. 736 of the State Council of the People’s Republic of China. http://www.gov.cn/zhengce/content/2021-01/29/content_5583525.htm; accessed 8 May 2023

[CR28] Coenen J, Bager S, Meyfroidt P, Newig J, Challies E (2021). Environmental governance of China's belt and road initiative. Environ Policy Gov.

[CR29] Denisia V (2010) Foreign direct investment theories: an overview of the main FDI theories. Eur J Interdiscip Stud. https://ssrn.com/abstract=1804514. Accessed 8 May

[CR30] Dong K, Sun W (2022). Would the market mechanism cause the formation of overcapacity?: evidence from Chinese listed firms of manufacturing industry. Int Rev Econ Finance.

[CR31] Dong Y, Li C (2021). China and the Reform of International Trade Governance System. Soc Sci China.

[CR32] Duarte CM, Bruhn A, Krause-Jensen D (2022). A seaweed aquaculture imperative to meet global sustainability targets. Nat Sustain.

[CR33] Dube K, Nhamo G, Chikodzi D (2021). COVID-19 cripples global restaurant and hospitality industry. Curr Issues Tour.

[CR34] ECCFIY (The Editor Committee of China Food Industry Yearbook) (1984–2020). China Food Industry Yearbook. China Statistics Press, Beijing

[CR35] ECCFSY (The Editor Committee of China Fishery Statistical Yearbook) (1979–2020) China Fishery Statistical Yearbook. China Agriculture Press, Beijing

[CR36] EFSA Panel on Food Additives and Nutrient Sources added to Food (ANS), Younes M, Aggett P, Aguilar F, Crebelli R, Filipič M, Frutos MJ, Galtier P, Gott D, Gundert-Remy U, Kuhnle GG, Lambré C, Leblanc JC, Lillegaard IT, Moldeus P, Mortensen A, Oskarsson A, Stankovic I, Waalkens-Berendsen I, Woutersen RA, Wright M, Brimer L, Lindtner O, Mosesso P, Christodoulidou A, Ioannidou S, Lodi F, Dusemund B (2018) Re‐evaluation of carrageenan (E 407) and processed *Eucheuma* seaweed (E 407a) as food additives. EFSA J 16:e05238

[CR37] FAO (2022) FAO Global Fishery and Aquaculture Production Statistics. https://www.fao.org/fishery/en/statistics/software/fishstatj/en; accessed 9 September 2022

[CR38] Ferdouse F, Holdt SL, Smith R, Murúa P, Yang Z (2018) The global status of seaweed production, trade and utilization. Globefish Res 124:I

[CR39] Fujian Huanyu Marine Biotechnology (2022) Construction project environmental impact report form: Fujian Huanyu Marine Biotechnology Co., Ltd.’s annual production of 500 tonnes of carrageenan, 100 tonnes of agar, and 100 tonnes of Shihua jelly. http://www.shishi.gov.cn/zwgk/zwgkzdgz/hjbh/jsxmhjyxpjxx/202201/W020220106415853084877.pdf; accessed 9 May 2023

[CR40] Fung D (2022) Producing in Multiple Countries Effectively Reduces Costs for Marine Science Firm: Interview with Freeman So, Chief Financial Officer and Secretary, Green Future Food Hydrocolloid Marine Science Co Ltd. HKDTC Research. https://research.hktdc.com/en/article/OTYxMDcxODMw; accessed 9 September 2022

[CR41] Ge GL, Ding DZ (2008). A strategic analysis of surging Chinese manufacturers: The case of Galanz. Asia Pac J Manag.

[CR42] Global petrol prices (2022) Electricity prices. https://www.globalpetrolprices.com/electricity_prices/#hl122; accessed 23 November 2022

[CR43] Grand View Research (2023). Carrageenan Market Size, Share & Trends Analysis Report by Processing Technology (Semi-refined, Gel Press, Alcohol Precipitation), By Function, By Product Type, By Application, By Region, And Segment Forecasts, 2023–2030.

[CR44] Green Future (2021) Annual report of Green Future Food Hydrocolloid Marine Science Company Limited. https://files.services/files/511/2022/0427/20220427171502_48563320_en.pdf ; accessed 7 November 2022

[CR45] Greenone Biotechnology (2023) Greenone Biotechnology Company Profile. http://www.greenone.hk/aboutus.html. Accessed 8 May 2023

[CR46] Guo SH (2013). Discussion on the bottleneck and countermeasures of carrageenan industry development in China (Qianyi kalajiao chanye fazhan pingjing jiqi duice). Chem Eng Equip.

[CR47] Ha J, Kose MA, Ohnsorge F (2022) Global Stagflation. 10.2139/ssrn.4131943

[CR48] Harrison-Dunn AR (2015) What will it take to make indonesian seaweed competitive? In: Food Navigator June 3. https://www.foodnavigator.com/Article/2015/06/03/What-will-it-take-to-make-Indonesian-seaweed-competitive. Accessed 22 Aug 2022

[CR49] Harvie C (1999) China's township and village enterprises and their evolving business alliances and organisational change. Department of Economics, University of Wollongong. https://ro.uow.edu.au/commwkpapers/9. Accessed 8 May 2023

[CR50] Hatch Seaweed Insights (2023a) Eucheumatoids. https://seaweedinsights.com/global-production-eucheumatoids/; accessed 12 May 2023a

[CR51] Hatch Seaweed Insights (2023b) Global Production Overview. https://seaweedinsights.com/global-production/; accessed 12 May 2023b

[CR52] Hermans S (2023) 2023 Seaweed State of the Industry. https://phyconomy.net/articles/2022-seaweed-review/; accessed 9 May 2023

[CR53] Hilliou L (2014). Hybrid carrageenans: isolation, chemical structure, and gel properties. Adv Food Nutr Res.

[CR54] Hotchkiss S, Brooks M, Campbell R, Philp K, Trius A (2016) The use of carrageenan in food. In: Pereira L (ed) Carrageenans: sources and extraction methods, molecular structure, bioactive properties and health effects. Nova Science Publishers, New York, pp 229–243

[CR55] Hurtado AQ, Critchley AT, Neish I (2017). Tropical seaweed farming trends, problems and opportunities.

[CR56] Hurtado AQ, Neish IC, Critchley AT (2019). Phyconomy: the extensive cultivation of seaweeds, their sustainability and economic value, with particular reference to important lessons to be learned and transferred from the practice of eucheumatoid farming. Phycologia.

[CR57] IFC (International Finance Corporation) (2007) Chinese Market for Seaweed and Carrageenan Industry. http://marineagronomy.org/sites/default/files/IFC%20-%20Final%20report%20-%20Chinese%20Market%20for%20Seaweed%20and%20Carrageenan%2028-04-07.pdf; accessed 10 May 2023

[CR58] JaSuDa (2022) JaSuDa.net: PT. Jaringan Sumber Daya. https://jasuda.net/; accessed 10 May 2023

[CR59] Jin S, Guo H, Delgado MS, Wang HH (2017). Benefit or damage? The productivity effects of FDI in the Chinese food industry. Food Policy.

[CR60] Kambey CS, Campbell I, Sondak CF, Nor AR, Lim PE, Cottier-Cook EJ (2020). An analysis of the current status and future of biosecurity frameworks for the Indonesian seaweed industry. J Appl Phycol.

[CR61] Kemenperin (2022). Kebijakan Industri Pengolahan Rumput Laut.

[CR62] Keohane A (2016) A look into the carrageenan industry: how tourism, markets and demand affect the seaweed farmers of Bali Indonesia. Center for Marine Biodiversity and Conservation, UC San Diego, California, pp 1–33. https://escholarship.org/uc/item/9pw032tn. Accessed 8 May 2023

[CR63] Kokko A (2006) The home country effects of FDI in developed economies. Working Paper No. 225. European Institute of Japanese Studies, Stockholm. https://citeseerx.ist.psu.edu/viewdoc/download?doi=10.1.1.509.4909&rep=rep1&type=pdf. Accessed 8 May 2023

[CR64] Komarek A, Cahyadi ER, Zhang J, Fariyanti A, Julianto B, Arsyi R, Lapong I, Langford A, Waldron S, Grist M (2022) Increasing incomes in carrageenan seaweed value chains in Takalar, South Sulawesi. Australia-Indonesia Centre, Melbourne, Victoria, pp 1–32

[CR65] Laborde D, Martin W, Swinnen J, Vos R (2020). COVID-19 risks to global food security. Science.

[CR66] Langford A, Turupadang W, Waldron S (2023) Interventionist industry policy to support local value-adding: Evidence from the Eastern Indonesian seaweed industry. Mar Policy 151:105561

[CR67] Langford A, Waldron S, Sulfahri SH (2021). Monitoring the COVID-19-affected Indonesian seaweed industry using remote sensing data. Mar Policy.

[CR68] Langford A, Zhang J, Waldron S, Julianto B, Siradjuddin I, Neish I, Nuryartono N (2022) Price analysis of the Indonesian carrageenan seaweed industry. Aquaculture 550:737828

[CR69] Lantern Today (2022) Satpol PP Kabupaten Madiun Panggil PT Newstar Konjak Nusantara, Ini Hasilnya. https://lenteratoday.com/satpol-pp-kabupaten-madiun-panggil-pt-newstar-konjak-nusantara-ini-hasilnya/; accessed 8 May 2023

[CR70] Liu Z, Mutukumira AN, Chen H (2019). Food safety governance in China: From supervision to coregulation. Food Sci Nutr.

[CR71] Longrun Newstar (2023) Longrun Newstar Food Group: About Us. http://www.longrun-newstar.com/index_en.php; accessed 8 May 2023

[CR72] Loureiro RR, Cornish ML, Neish IC (2017) Applications of carrageenan: with special reference to iota and kappa forms as derived from the eucheumatoid seaweeds. In: Hurtado AQ, Critchley AT, Neish I (eds) Tropical Seaweed Farming Trends, Problems and Opportunities: Focus on *Kappaphycus* and *Eucheuma* of Commerce. Springer, Cham, pp 165–171

[CR73] Mariño M, Breckwoldt A, Teichberg M, Kase A, Reuter H (2019) Livelihood aspects of seaweed farming in Rote Island, Indonesia. Mar Policy 107:103600

[CR74] McHugh DJ (2003) A guide to the seaweed industry. FAO Fisheries Technical Paper 441. Food and Agriculture Organization of the United Nations, Rome, pp 1–105

[CR75] McVey RT (1992) Southeast Asian Capitalists (No. 9). Cornell University Press, Southeast Asia Program Publications at Cornell University. https://www.jstor.org/stable/; accessed 10 May 2023

[CR76] Media Indonesia (2019) Petani Rumput Laut Rugi Akibat Limbah Tambang Nikel (Seaweed Farmers Lose Due to Nickel Mining Waste). Media Indonesia, 30 June 2019. https://mediaindonesia.com/nusantara/244206/petani-rumput-laut-rugi-akibat-limbah-tambang-nikel; accessed 2 February 2023

[CR77] Media Indonesia (2022) Ministry of Environment and Forestry Asked to Test River Water at PT Biota Laut Ganggang Wastewater Disposal Site (KLHK Diminta Lakukan Pengujian Air Sungai Tempat Pembuangan Limbag PT Biota Laut Ganggang). https://mediaindonesia.com/humaniora/528588/klhk-diminta-lakukan-pengujian-air-sungai-tempat-pembuangan-limbag-pt-biota-laut-ganggang; accessed 2 February 2023

[CR78] Mulyati H, Geldermann J, Kusumastanto T (2020) Carrageenan supply chains in Indonesia. IOP Conf Ser: Earth Environ Sci 414:012013

[CR79] NBSC (National Bureau of Statistics of China) (2021) China Statistical Yearbook. http://www.stats.gov.cn/tjsj/ndsj/2021/indexch.htm; accessed 23 January 2023

[CR80] NDRC (National Development and Reform Commission) (2019) Catalogue for Guiding Industry Restructuring (2019 version). http://www.gov.cn/gongbao/content/2020/content_5467513.htm; accessed 18 August 2022

[CR81] Necas J, Bartosikova L (2013) Carrageenan: a review. Vet Med (Praha) 58:187–205

[CR82] Neish IC (2015) A diagnostic analysis of seaweed value chains in Sumenep Regency, Madura Indonesia. United Nations Industrial Development Organization, Vienna. UNIDO 140140:1–66

[CR83] Neish IC (2020) Ten success factors for the coral triangle seaweed industry. Video presented at the 10th International Seaweed Conference, 15–16 September 2021. YouTube: https://www.youtube.com/watch?v=hUzMaL7vTg0&t=373s; accessed 8 May 2023

[CR84] Neish IC, Sepulveda M, Hurtado AQ, Critchley AT (2017) Reflections on the commercial development of eucheumatoid seaweed farming. In: Hurtado AQ, Critchley AT, Neish I. (eds) Tropical Seaweed Farming Trends, Problems and Opportunities: Focus on *Kappaphycus* and *Eucheuma* of Commerce. Springer, Cham, pp 1–27

[CR85] NHC (National Health Commission of the PRC) (2016) National Food Safety Standard for Food additive – Carrageenan (GB 1886.171–2016). http://www.gd-sct.com/dgweb_content-876117.html; accessed 10 May 2023

[CR86] Ningrum DR (2016) Mapping of Policies and Stakeholders in Foreign Direct Investment in Indonesia Agriculture Sector. Perkumpulan PRAKARSA. https://repository.theprakarsa.org/publications/293904/mapping-of-policies-and-stakeholders-in-foreign-direct-investment-in-indonesia-a; accessed 10 May 2023

[CR87] Novaczek I, Harkes I, Sopacua J, Tatuhey M (2001) An institutional analysis of sasi laut in Maluku, Indonesia. ICLARM-The World Fish Center, Penang, Malaysia. ICLARM Tech Rep (59) pp 1–327. https://hdl.handle.net/20.500.12348/2333; accessed 10 May 2023

[CR88] NPC (National People's Congress) (2019) Foreign Investment Law of the People’s Republic of China. Order of the President of the People's Republic of China No. 26. http://www.npc.gov.cn/zgrdw/npc/xinwen/2019-03/15/content_2083532.htm; accessed 8 May 2023

[CR89] Nuryartono N, Waldron S, Langford A, Sulfahri, Tarman K, Pasaribu SH, Siregar UJ, Lusno MFD (2020) A diagnostic analysis of the South Sulawesi seaweed industry. Australia-Indonesia Centre, Melbourne.

[CR90] Olatunji O (2020) Carrageenans. In: Olatunji O (ed) Aquatic Biopolymers: Understanding their Industrial Significance and Environmental Implications. Springer, Cham, pp 121–144

[CR91] Ozili PK, Arun T (2023) Spillover of COVID-19: impact on the global economy. In: Akkucuk U (ed) Managing Inflation and Supply Chain Disruptions in the Global Economy. IGI Global, Hershey, Pennsylvania, pp 41–61

[CR92] Palanca-Tan R (2018). Aquaculture, poverty and environment in the Philippines. J Soc Political Econ Stud.

[CR93] Pei X, Tandon A, Alldrick A, Giorgi L, Huang W, Yang R (2011). The China melamine milk scandal and its implications for food safety regulation. Food Policy.

[CR94] Pereira L (2018) Biological and therapeutic properties of the seaweed polysaccharides. Int Biol Rev 2:1–50

[CR95] Porse H, Rudolph B (2017). The seaweed hydrocolloid industry: 2016 updates, requirements, and outlook. J Appl Phycol.

[CR96] Presidential Regulation No 33 (2019) Road Map for Development of National Seaweed Industry In 2018–2021 (Peraturan Presiden Nomor 33 Tahun 2019 tentang Peta Panduan Pengembangan Industri Rumput Laut Nasional Tahun 2018-2021). https://jdih.kkp.go.id/Homedev/DetailPeraturan/93#. Accessed 8 May 2023

[CR97] Qin Y (2018) Seaweed bioresources. In: Qin Y (ed) Bioactive Seaweeds for Food Applications: Natural Ingredients for Healthy Diets. Academic Press, New York, pp 3–24

[CR98] Ren N, Liu H (2022). Southeast Asian Chinese engage a rising China: business associations, institutionalised transnationalism, and the networked state. J Ethn Migr Stud.

[CR99] Richards-Rajadurai N (1990) Production, marketing and trade of seaweeds. In: Technical Resource Papers Regional Workshop on the Culture and Utilization of Seaweeds Volume II. Food and Agriculture Organization of the United Nations https://www.fao.org/3/ab728e/AB728E12.htm; accessed 28 April 2023

[CR100] Rieve K (2023) Are investors in the seaweed sector looking in the wrong place? https://thefishsite.com/articles/are-investors-in-the-seaweed-sector-looking-in-the-wrong-place-hatch-seaweed-insights; accessed 9 May 2023

[CR101] SAMR (State Administration for Market Regulation) (2020) Measures for the Administration of Food Production Licensing. Order No. 24 of the State Administration for Market Regulation. https://gkml.samr.gov.cn/nsjg/fgs/202001/t20200103_310238.html; accessed 8 May 2023

[CR102] Saragih AK, Burhanuddin B, Herawati H (2022) Determinant analysis of Indonesian seaweed trade. J Integr Agribus 4:77–87

[CR103] Shi D, Yang Z, Ji H (2022) Energy target-based responsibility system and corporate energy efficiency: Evidence from the eleventh Five Year Plan in China. Energy Policy 169:113214

[CR104] Shi Y, Cheng C, Lei P, Wen T, Merrifield C (2011). Safe food, green food, good food: Chinese Community Supported Agriculture and the rising middle class. Int J Agric Sustain.

[CR105] Skinner GW (1963) The Chinese Minority. In McVey RT (ed) Indonesia, Survey of World Cultures 12. Yale University Press, New Haven, Connecticut, pp 97–117

[CR106] Stanley N (1987) Production, properties and uses of carrageenan. In: McHugh DJ (ed) Production and utilization of products from commercial seaweeds. FAO Fisheries Technical Paper. https://www.fao.org/3/x5822e/x5822e05.htm. Accessed 23 Nov 2022

[CR107] Sudarwati W, Hardjomidjojo H, Setyaningsih D (2020) Literature review: potential and opportunities for the development of seaweed agro-industry. IOP Conf Ser: Earth Environ Sci 472:012063

[CR108] Sumule O, Angkasa WI, Retno HW, Andiewati S (2021) The development of Indonesian seaweed based on innovation cluster model. IOP Conf Ser: Earth Environ Sci 763:012016

[CR109] Thomas WR (1997) Carrageenan. In: Imeson AP (ed) Thickening and Gelling Agents for Food. Springer, Boston, MA, pp 45–59

[CR110] Tritto A (2021) China's Belt and Road Initiative: from perceptions to realities in Indonesia's coal power sector. Energy Strategy Rev 34:100624

[CR111] UN Comtrade (United Nations Statistics Division) (2022) International Merchandise Trade Statistics. http://comtrade.un.org/. Accessed 10 May 2023

[CR112] Utomo BSB, Mulia RAY (2013). The quality of alkali treated cottonii (ATC) made from Eucheuma cottonii collected from different regions in Indonesia. Squalen Bull Mar Fish.

[CR113] Valderrama D, Cai J, Hishamunda N, Ridler N (2013) Social and economic dimensions of carrageenan seaweed farming. FAO Fish Aquacult Tech Paper 580:1–217

[CR114] Valderrama D, Cai J, Hishamunda N, Ridler N, Neish IC, Hurtado AQ, Msuya FE, Krishnan M, Narayanakumar R, Kronen M, Robledo D (2015). The economics of *Kappaphycus* seaweed cultivation in developing countries: a comparative analysis of farming systems. Aquacult Econ Manag.

[CR115] Wang X, He L, Ma Y, Huan L, Wang Y, Xia B, Wang G (2020) Economically important red algae resources along the Chinese coast: History, status, and prospects for their utilization. Algal Res 46:101817

[CR116] Ward GM, JrJP F, Cottier-Cook EJ, Gachon C, Hurtado AQ, Lim PE, Matoju I, Msuya FE, Bass D, Brodie J (2020). A review of reported seaweed diseases and pests in aquaculture in Asia. J World Aquac Soc.

[CR117] Waters TJ, Lionata H, Prasetyo Wibowo T, Jones R, Theuerkauf S, Usman S, Amin I, Ilman M (2019) Coastal conservation and sustainable livelihoods through seaweed aquaculture in Indonesia: A guide for buyers, conservation practitioners, and farmers. The Nature Conservancy. Arlington VA, USA and Jakarta, Indonesia. https://www.nature.org/content/dam/tnc/nature/en/documents/Indonesia_Seaweed_Guide_FINAL.pdf; accessed 10 May 2023

[CR118] Webb EJ, Campbell DT, Sechrest L, Schwartz R (2000). Unobtrusive Measures.

[CR119] Weber M, Gorodnichenko Y, Coibion O (2023). The expected, perceived, and realized inflation of us households before and during the covid19 pandemic. IMF Econ Rev.

[CR120] Wilkinson J (2008) The food processing industry, globalization and developing countries. In: McCullough EB, Pingali PL, Stamoulis KG (Eds) The transformation of agri-food systems: globalization, supply chains and smallholder farmers. Food & Agriculture Organization, London, Sterling, VA, pp 87–108.

[CR121] Wiratmini NPE (2018) Produksi Rumput Laut di Bali Anjlok 99% (Seaweed Production in Bali Plummets 99%). Ekonomi, March 8, 2018. Available at: https://ekonomi.bisnis.com/read/20180308/99/747619/produksi-rumput-laut-di-bali-anjlok-99; accessed 2 February 2023

[CR122] Woodworth MD, Ren X, Rodenbiker J, Santi E, Tan Y, Zhang L, Zhou Y (2022) Researching China during the COVID-19 pandemic. In: Brunn SD, Gilbreath D (eds) COVID-19 and a World of Ad Hoc Geographies. Springer, Cham, pp 2705–2721

[CR123] Wright EC (2017) The Upshot Of Upgrading: Seaweed Farming And Value Chain Development In Indonesia . Doctoral dissertation, University of Hawaiʻi at Mānoa. https://www.proquest.com/dissertations-theses/upshot-upgrading-seaweed-farming-value-chain/docview/1954699097/se-2; accessed 9 September 2022

[CR124] Yamin D, Tarbuck P (2021) Rebalancing the Indonesian seaweed industry. Economist Impact’s World Ocean Initiative. https://ocean.economist.com/innovation/articles/rebalancing-the-indonesian-seaweed-industry; accessed 20 November 2022

[CR125] Yang Y, Xu H, Zhang Y, Guo X (2023) The evolution of China's environmental impact assessment system: Retrospect and prospect from the perspective of effectiveness evaluation. Environ Impact Assess Rev 101:107122

[CR126] Yao H, Zhong M, Liu W, Chen B (2022). Belt and Road Initiative and OFDI from China: the paradox of home country institutional environment between state and local governments. Chin Manag Stud.

[CR127] Yemenicioğlu A, Farris S, Turkyilmaz M, Gulec S (2020) A review of current and future food applications of natural hydrocolloids. Int J Food Sci Technol 55:1389–1406. 10.1111/ijfs.14363

[CR128] Zhang Q, Duan D (2021) Present status and trends of carrageenan production in China. Video presented at the Tropical Phyconomy Coalition Development Online Workshop. YouTube. https://www.youtube.com/watch?v=ttdjD4lRL70; accessed 10 May 2023

